# Multiphase Coexistence in Binary Hard Colloidal Mixtures: Predictions from a Simple Algebraic Theory

**DOI:** 10.1021/acs.jpclett.2c03138

**Published:** 2022-12-29

**Authors:** J. Opdam, V. F. D. Peters, H. H. Wensink, R. Tuinier

**Affiliations:** † Laboratory of Physical Chemistry, Department of Chemical Engineering and Chemistry, and Institute for Complex Molecular Systems (ICMS), Eindhoven University of Technology, P.O. Box 513, 5600 MBEindhoven, The Netherlands; ‡ Department of Earth Sciences, Utrecht University, Princetonlaan 8a, 3584CBUtrecht, The Netherlands; § Laboratoire de Physique des Solides, Université Paris-Saclay and CNRS, 91405Orsay, France

## Abstract

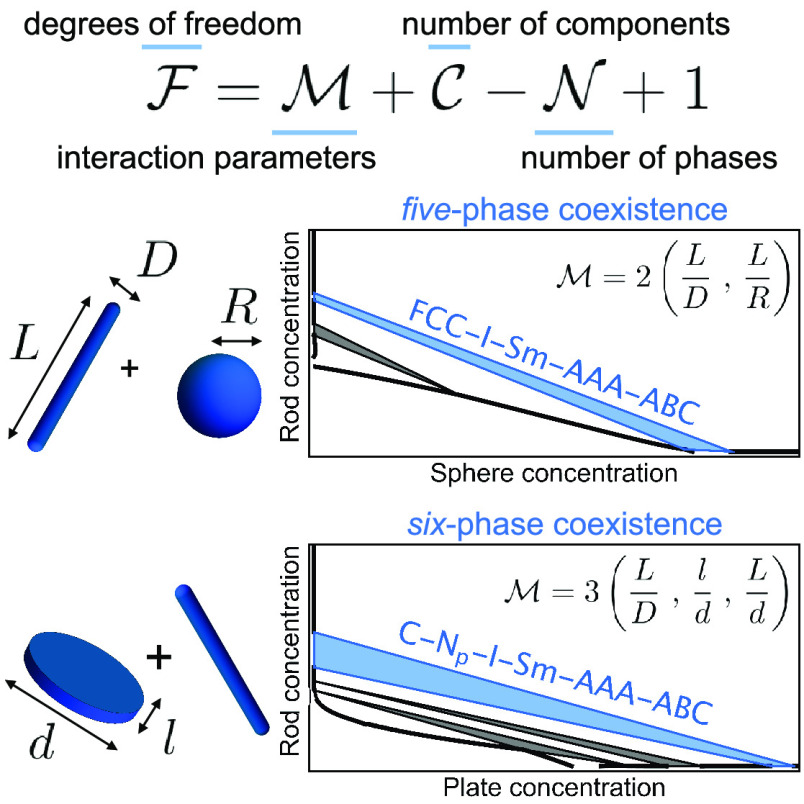

A general theoretical framework is proposed to quantify the thermodynamic properties of multicomponent hard colloidal mixtures. This framework is used to predict the phase behavior of mixtures of rods with spheres and rods with plates taking into account (liquid) crystal phases of both components. We demonstrate a rich and complex range of phase behaviors featuring a large variety of different multiphase coexistence regions, including two five-phase coexistence regions for hard rod/sphere mixtures, and even a six-phase equilibrium for hard rod/plate dispersions. The various multiphase coexistences featured in a particular mixture are in line with a recently proposed generalized phase rule and can be tuned through subtle variations of the particle shape and size ratio. Our approach qualitatively accounts for certain multiphase equilibria observed in rod/plate mixtures of clay colloids and will be a useful guide in tuning the phase behavior of shape-disperse mixtures in general.

Multicomponent colloidal dispersions containing anisotropic particles exhibit fascinating phase behavior.^1−14^ In the case of pure hard-core particle interactions, the phase behavior of plate-like^15,16^ or rod-shaped^17,18^ colloids is much richer than that of their spherical counterparts.^19,20^ While at low particle concentrations such dispersions are fluidic, a hard sphere fluid transforms into a solid phase state at high concentrations. A dispersion of hard plates can exhibit a liquid crystalline nematic phase as well as a solid-like columnar phase,^15,16^ and hard rods can also assume liquid crystalline phase states such as nematic^21^ and smectic phases^22,23^ in addition to different types of solid phases.^17^ Dispersions containing two or more colloidal particles with different shapes thus have a wide variety of possible phase coexistences. The richness of the phase behavior was demonstrated experimentally in a model colloidal rod/plate mixture by Van der Kooij and Lekkerkerker,^3,24^ who observed a variety of three-phase and four-phase coexistences and even a five-phase coexistence. Such coexistences are very challenging to reproduce in numerical simulations; however, with a theoretical approach, the multiphase coexistence behavior of colloidal mixtures can be qualitatively predicted in a quick and relatively easy manner as shown in this Letter.

Predicting the phase behavior is of practical importance because colloidal multicomponent mixtures are ubiquitous in products such as paint^25^ and food^26^ and are present in natural systems such as opals^27^ and crowded living cells,^28^ where phase transitions play an important role in several intracellular processes.^29,30^ Knowledge of colloidal phase behavior is also a key input for the development of novel applications such as high-performance photovoltaic materials,^31^ energy storage materials,^32^ and novel types of batteries^33^ and fuel cells.^34^ The wide variety of phases emerging in colloidal mixtures makes them of potential interest for the fabrication of phase-change materials.^35^ Moreover, excluded volume interactions have been used to generate novel structures such as superlattices^36^ or quasi-crystals.^37^ Insights into colloidal phase behavior can also be of use for the separation or purification of colloidal components such as bacteria,^38^ viruses,^39^ clay,^10,40^ or nanoparticles^41,42^ in complex mixtures.

Here, we present a simple and general framework that enables one to compute the phase behavior of colloidal mixtures, including anisotropic particles, and we apply this to the case of rods (spherocylinders) mixed with spheres and also show results for binary mixtures of platelets (disks) and rods. The phase behavior of binary mixtures of colloidal particles has been investigated previously with a variety of theoretical methods such as density functional theory (DFT),^43,44^ Parsons–Lee theory (PL),^4,12^ and free volume theory (FVT).^14,45,46^ However, these studies include positionally ordered phases of only one component. This imposes a limitation on the range of applicability with regard to size ratios and concentrations and does not allow computation of the coexistence between a phase in which one component exhibits positional order and a phase in which the other component exhibits positional order. In this study, we overcome this limitation and include phases in which either one of the components is allowed to adopt orientational and positional ordered states and we showcase a wide range of possible multiphase coexistences that can be reconfigured by tuning the principal size ratios of the mixture.

With the general theoretical method outlined in this Letter, it is not possible to account for phase states in which both components are ordered simultaneously. It is important to point out that our phase diagrams consistently feature strong demixing and fractionation effects and the phase states coexisting at any of the multiphase equilibria are all strongly enriched in one component while the other species is present at only relatively small volume fractions. Under such conditions, the orientational or positional order of the minor component will have an only minor influence on the free energy of any phase state of the binary mixture. In dense mixtures with different types of anisotropic particles, there are likely conditions under which both components remain well-mixed and generate structures in which both components are ordered. Theoretically, these cases could be addressed using DFT (see ref (47) for a discussion on dense multicomponent hard particle fluids), although treating both components as freely rotating objects poses serious technical difficulties.^48,49^ In the future, it will be of interest to compare the relative stability of phase states in which both components exhibit order with respect to the (multiphase) demixing regions discussed here.

The colloidal particles we consider are monodisperse and interact solely through “hard” excluded volume interactions. In recent decades, developments in colloid synthesis^50^ enabled the preparation of well-defined hard colloidal particles of a wide range of shapes.^51,52^ Although colloids in complex industrial or biological systems typically interact through various types of forces, excluded volume interactions are usually a prevalent type of interaction and become increasingly important in dense systems. Although additional interactions and particle polydispersity influence colloidal phase behavior, it is expected that the trends discussed in this Letter also hold for more complex size- and shape-disperse systems.

We consider a canonical ensemble and define the Helmholtz free energy *F* of an athermal binary mixture of hard colloidal particles as a perturbation of the free energy of a one-component system :
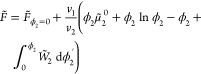
1with ϕ_*i*_ is the volume fraction, *v*_*i*_ is the volume, μ_*i*_ is the chemical potential, *W*_*i*_ is the work of insertion of colloidal component *i*, and the constant , where  is the de Broglie wavelength. The free energy of a system containing only component 1 contains both ideal and non-ideal contributions: . The used normalized quantities are , , , and , with *k*_B_ is the Boltzmann constant, *T* the temperature, and *V* the volume of the system. A concise derivation of eq 1 is shown in section 1 of the Supporting Information.

It is important to note that the Widom expression^53^ is used for the chemical potential of component 2, which means that component 2 is assumed to behave as a fluid phase with no orientational or translational order. This means that, using this approach, it is not possible to account for binary crystals or biaxial phases in which both components are ordered. However, the free energy of a dense phase where particles of component 1 are ordered and component 2 behaves as a fluid can be determined with eq 1, and the same equation can be used for the free energy of a dense phase where particles of component 2 are ordered and component 1 behaves as a fluid, simply by interchanging the subscripts “1” and “2”.

The chemical potential of both components in a certain phase and the osmotic pressure of the corresponding phase are determined with the following standard thermodynamic expressions: , , and . Coexistence curves are determined by solving the coexistence conditions:
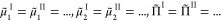
2where the subscripts denote either component 1 or 2, the superscripts denote a certain phase state, and the dots indicate that the same equations are used for multiphase coexistence regions. The free energy expression given by eq 1 can also be extended to describe mixtures of more than two components, as shown in section 2 of the Supporting Information, and a similar expression has been used to incorporate size polydispersity into the free energy expression for colloid/polymer mixtures.^54,55^

In section 3 of the Supporting Information, it is shown that eq 1 is the canonical equivalent to the expression used for the semi-grand canonical potential of a colloidal mixture in FVT.^14,46,56^ This method has recently been used to study phase boundaries in binary mixtures of hard spheres^56^ and hard rod/sphere mixtures.^14,46^ In these works, ordered phases of only one component were accounted for whereas the depletants are treated as a fluid with a constant chemical potential that is fixed by an external reservoir. Because we are interested in mapping the phase behavior of colloidal mixtures in which both particles can form ordered phases, a canonical ensemble, without the need to use an external reservoir, is used here. The FVT predictions were shown to be in qualitative and reasonable quantitative agreement with computer simulation and experimental results, which is an indication of the validity of eq 1 in the parameter range of these studies. In the future, a detailed comparison between the predicted variety of possible phase coexistences presented in this Letter and an overview of computer simulation results and experimental observations on various colloidal mixtures is desirable.

Defining the free energy of the binary system as a perturbation on the free energy of a one-component system (eq 1) allows us to account for phases displaying the translational order of either component. Using eq 1 is, however, possible only if the free energies of the considered phases are known for the one-component colloidal systems (ϕ_2_ = 0) of interest. A variety of theoretical methods for obtaining accurate approximations for the equations of state of one-component systems such as DFT,^57,58^ PL,^59,60^ scaled particle theory (SPT),^61−63^ virial expansions,^64,65^ and Lennard-Jones–Devonshire (LJD) cell theory are available.^66−68^Table 1 shows an overview of the different colloidal mixtures and phases studied in this Letter. Also indicated are the theoretical methods used as input for applying eq 1. For each phase, one type of particle is identified as component 1 and an appropriate free energy expression for the one-component system  is chosen on the basis of a specific theoretical approximation for an equation of state. Component 2 is considered to behave as a fluid and is included through Widom insertion theory.^53^ The expressions used for  in the calculations are outlined in section 4 of the Supporting Information.

**Table 1:** Overview of the Colloidal Mixtures and Phases Considered in This Letter and the Theoretical Methods Used as Input for eq 1^a^

rods and spheres			rods and plates		
phase	rods	spheres	phase	rods	plates
ABC	LJD^18^	W	ABC	LJD^18^	W
AAA	LJD^18^	W	AAA	LJD^18^	W
Sm	LJD^18^	W	Sm	LJD^18^	W
N	SPT^62^	W	N_R_	SPT^62^	W
I	W	CS^69^	I	W	PL^70^
FCC	W	LJD^66^	N_p_	W	PL^70^
			C	W	LJD^70^

a The principal phases are AAA and ABC crystal phases in which rods are ordered in a lamellar fashion with hexagonal order in each layer, with rods stacked on top of each other and between rods of adjacent layers, respectively. In smectic A phase (Sm), rods are ordered in a lamellar fashion but there is no translational order within each layer. The nematic (N) phase has orientational order along the nematic director but no translational order. An isotropic phase in which no component displays orientational or translational order is phase I. A face-centered cubic crystal of spheres (FCC) and a columnar phases of plates (C). CS refers to the Carnahan–Starling equation of state for a fluid of hard spheres, and W refers to Widom insertion theory.^53^

We make use of SPT to estimate the work of insertion  for a particle of component 2 into the colloidal mixture, which requires the volume, surface area, and integrated mean curvature of both colloidal components as input parameters. It is noted that this approach to determine the work of insertion does not account for any orientational or positional order of component 1, which likely results in an underestimation of the work of insertion at low concentrations and an overestimation at high concentrations for ordered phases.^71^ Although there are ways to improve the expressions for  for specific ordered phases containing particles with certain shapes,^56,70,72^ there is no general theoretical method for determining the work of insertion in ordered phases, to the best of our knowledge. A summary of the general SPT method employed in this Letter for obtaining expressions for  is given in section 5 of the Supporting Information.

First, we study the phase behavior of mixtures of colloidal hard rods and hard spheres. The rods are modeled as spherocylinders with length *L*_r_ and diameter *D*_r_. The hard sphere diameter equals *D*_s_. Figure 1a shows an overview of multiphase coexistence regions found in phase diagrams of mixtures of colloidal rods and spheres. Such phase diagrams can generally be characterized by different types of three-phase coexistence regions (triple) that span the phase diagram. The areas in Figure 1a, indicated with Roman numbers, highlight regions in the parameter space where phase diagrams with a specific set of triple regions can be found. Illustrative phase diagrams for each region indicated in Figure 1a are shown in section 6 of the Supporting Information. The locations of the different triple regions in the phase diagrams of rod/sphere mixtures strongly depend on the *D*_s_/*D*_r_ size ratio and *D*_r_/*L*_r_ (inverse) aspect ratio. Changing the size ratio or the rod aspect ratio can thus result in two contiguous triple regions, leading to a quadruple region. The curves, marked by numbers in brackets, indicate the parameters for which coexistence among four different phases can occur. An overview of the different three-phase and four-phase coexistence regions found in Figure 1a is given in Table 2. Coexistence regions that contain two phases with the same structure but different concentrations, such as N–I–I–FCC coexistence, are not shown in the overview of Figure 1a or Table 2.

**Figure 1:**
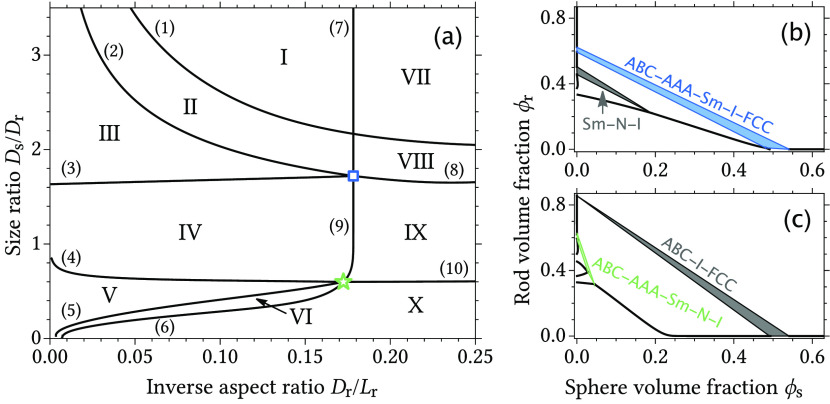
(a) Overview of the types of multiphase coexistence regions (excluding isostructural phases) predicted for hard rod/sphere mixtures shown in size ratio *D*_s_/*D*_r_ and inverse aspect ratio *D*_r_/*L*_r_ representations. The curves indicate the parameters at which four-phase coexistence is found. The type of four-phase coexistence region corresponding to each curve is indicated in Table 2. The Roman numbers indicate the regions between the four-phase coexistence curves in which phase diagrams with a certain topology, as shown in Table 2, are found. The symbols denote the parameters for which five-phase ABC–AAA–Sm–I–FCC (square symbol, *D*_r_/*L*_r_ = 0.178, *D*_s_/*D*_r_ = 1.72) and ABC–AAA–Sm–N–I (star symbol, *D*_r_/*L*_r_ = 0.172, *D*_s_/*D*_r_ = 0.598) coexistence regions are found. Phase diagrams containing the quintuple phase coexistence regions are shown in panels b and c.

**Table 2:** Overview of Three-Phase Coexistence Regions and Four-Phase Coexistence Regions (excluding isostructural phases) in Colloidal Mixtures of Spheres and Rods as Presented in the Phase Overview in Figure 1a*

	quadruple region		triple regions
1	Sm–N–I–FCC	I	N–I–FCC → Sm–N–FCC → AAA–Sm–FCC → ABC–AAA–FCC
2	AAA–Sm–I–FCC	II	Sm–N–I → Sm–I–FCC → AAA–Sm–FCC → ABC–AAA–FCC
3	ABC–AAA–I–FCC	III	Sm–N–I → AAA–Sm–I → AAA–I–FCC → ABC–AAA–FCC
4	AAA–Sm–N–I	IV	Sm–N–I → AAA–Sm–I → ABC–AAA–I → ABC–I–FCC
5	ABC–AAA–N–I	V	AAA–Sm–N → AAA–N–I → ABC–AAA–I → ABC–I–FCC
6	ABC–AAA–Sm–N	VI	AAA–Sm–N → ABC–AAA–N → ABC–N–I → ABC–I–FCC
7	ABC–AAA–Sm–FCC	VII	N–I–FCC → Sm–N–FCC → ABC–Sm–FCC
8	ABC–Sm–I–FCC	VIII	Sm–N–I → Sm–I–FCC → ABC–Sm–FCC
9	ABC–AAA–Sm–I	IX	(ABC–AAA–Sm →) Sm–N–I → ABC–Sm–I → ABC–I–FCC
10	ABC–Sm–N–I	X	(ABC–AAA–Sm →) ABC–Sm–N → ABC–N–I → ABC–I–FCC

* The order of the three-phase coexistence regions indicates the order in which the three-phase coexistence regions are present in the phase diagram going from low to high total particle concentrations. The phases denoted in the middle of the three-phase and four-phase coexistences are no longer present in the phase diagram at concentrations above the multiphase coexistence region. The AAA phase occurs when *D*_r_/*L*_r_ ≲ 0.18, which is where the ABC–AAA–Sm triple point occurs for a pure hard rod system.

The size ratio at which four-phase coexistence is found depends on the rod aspect ratio. For two specific sets of parameters, indicated by the star and square symbols, quadruple curves intersect, resulting in the possibility of coexistence among five different phases. The phase diagrams corresponding to these particular quintuple regions are shown in panels b and c of Figure 1. The possibility of five coexisting phases in a binary mixture of hard particles is counterintuitive because the maximum number of coexisting phases is only three according to the Gibbs phase rule for athermal systems: , where  is the number of degrees of freedom,  the number of components in the system, and  the number of coexisting phases. The term +1 can be replaced with +2 to describe systems with variable temperature. Recently, a generalized Gibbs phase rule was proposed to account for additional parameters^35,73,74^ that affect the phase behavior:
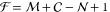
3where  is the number of independent variables affecting the phase behavior. This generalized Gibbs phase rule justifies the possibility of five-phase coexistence in a hard rod/sphere mixture because the rod aspect ratio and sphere/rod size ratio influence the phase behavior. Eq 3 follows naturally from the coexistence conditions in eq 2. The chemical potential of each component should be equal in all coexisting phases, and the osmotic pressure of all phases has to be equal, resulting in  equations that have to be solved to obtain coexistence densities. The number of variables needed is thus , where the number of parameters corresponding to the volume fraction of each component in each phase is given by  and  are the number of additional parameters needed to solve the coexistence equations. Every free parameter that influences the phase behavior thus allows for the possibility of an additional phase in multiphase coexistence regions. Therefore, even though the shapes and sizes of particles are usually not variable parameters in conventional colloidal systems, there are specific values for size ratios and shape parameters where additional phases meet the coexistence conditions and coexistences among more than three phases are theoretically possible even in athermal two-component systems.

The bottom part of Figure 1a, where the spheres are relatively small compared to the rods, is very similar to the phase stability overview of rod/polymer mixtures previously reported by Peters et al.^74^ In their work, they also reported a stable ABC–AAA–Sm–N–I quintuple phase coexistence region, although at a slightly lower *D*_s_/*D*_r_ size ratio and *D*_r_/*L*_r_ inverse rod aspect ratio. For small spherical depletants, the excluded volume between the spheres themselves does not strongly affect the phase behavior because phase transitions already occur at very low sphere concentrations, and therefore, one can expect that the phase behavior of rod/sphere mixtures and rod/polymer mixtures is similar in this region. When the spheres become large with respect to the rods, the phase where the spheres are ordered in an FCC crystal already appear at relatively low rod volume fractions. The four-phase coexistence curves (1)–(3) and (7)–(8) involve the ordered FCC phase and therefore do not occur in rod/polymer mixtures.

Another aspect in which the phase behavior of binary colloidal mixtures significantly deviates from that of colloid/polymer mixtures is the stability region of isotropic fluid–fluid (I–I) coexistence. A long-range interaction is generally needed to stabilize I–I equilibria. Therefore, in colloid/polymer mixtures, I–I coexistence generally occurs only above a certain relative polymer size and the extent of the I–I coexistence region (the liquid window) increases with relative polymer size.^75^ For binary colloidal mixtures, this does not hold because ordered phase states are expected whenever one of the particles is much larger than the other particles. Hence, we find that a certain anisotropy in particle shape is needed to induce a depletion interaction with a sufficient strength and range to obtain a stable I–I coexistence region in a binary colloidal mixture. For binary hard sphere mixtures, such I–I coexistence is metastable for all size ratios.^56,76^

Figure 2 shows phase diagrams for a hard rod/sphere mixture with an increasing relative rod length from panel a to panel e. For the relatively shortest rods shown here (*L*_r_/*D*_r_ = 37.5), I–I coexistence is metastable (dashed curve in panel a) with respect to I–FCC coexistence. At a certain composition, the fluid branches of the N–I and I–FCC coexistence curves meet, which denotes the onset of a triple N–I–FCC coexistence region. This triple region is bordered by the I–FCC, N–I, and N–FCC two-phase coexistence regions at relatively low rod concentrations, low sphere concentrations, and high total concentrations, respectively. When the relative rod length is increased to *L*_r_/*D*_r_ = 50, there is a small stable region of I–I coexistence and an I–I–FCC three-phase coexistence region at concentrations below the N–I–FCC coexistence region. With an increase in the relative rod length to *L*_r_/*D*_r_ = 53.5, the I–I–FCC coexistence region connects with the N–I–FCC coexistence region and a four-phase N–I–I–FCC region is found. For larger *L*_r_/*D*_r_ values, the I–I region is no longer connected to the solid FCC phase, but instead, an N–I–I triple region appears. Finally, for very long rods (*L*_r_/*D*_r_ = 93.8), the I–I region becomes metastable again but this time with respect to N–I coexistence.

**Figure 2:**
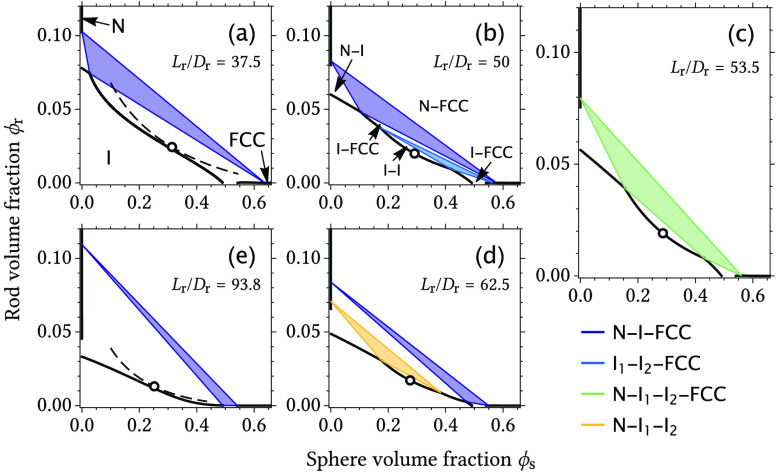
Phase diagrams of binary mixtures of hard rods and spheres with an increasing rod aspect ratio from panel a to panel e. The *D*_s_/*D*_r_ sphere/rod size ratio is 12.5. (c) Aspect ratio at which a four-phase nematic–isotropic–isotropic–FCC (N–I–I–FCC) coexistence region is found. The symbols denote the I–I critical points, and the dashed curves in panels a and e indicate that the I–I coexistence is metastable with respect to I–FCC and N–I, respectively. The Sm, AAA, and ABC phases have been omitted here because these phases occur at relatively high rod concentrations for the considered rod aspect ratios.

The phase diagrams in Figure 2 show that a stable I–I region is found for only a narrow range of rod aspect ratios, whereas the I–I phase becomes metastable to either I–FCC phase coexistence for short rods or N–I coexistence for long rods. Furthermore, the N–I and I–FCC coexistence regions become very small for short rods and long rods, respectively, as seen in panels a and e. The largest liquid window is found exactly at the rod aspect ratio where the four-phase N–I–I–FCC coexistence region is found (see Figure 2c).

Next, we discuss the multiphase coexistence behavior of colloidal mixtures containing two anisotropic particles: rods and plates. The platelets are described as hard disks with a thickness *L*_p_ and a diameter *D*_p_. The phase behavior of rods and plates has been studied previously with a focus on a specific set of parameters^5,6,43,77^ or on the stability region of a specific phase coexistence.^78^ An illustrative phase diagram for a mixture of hard rods and hard plates, including phases with translational ordering, is shown in Figure 3a. The rod and disk aspect ratios are as follows: *L*_r_/*D*_r_ = 10, and *L*_p_/*D*_p_ = 1/15, respectively. The size ratio between the particles (*L*_r_/*D*_p_) is set to 4/3. Similar to colloidal mixtures of rods and spheres, demixing into strongly fractionated phases occurs when the concentration of rods or plates increases and the phase diagrams can be characterized by a number of three-phase coexistence regions that span the phase diagram. The slope of these regions and the coexistence tie-lines depends strongly on the size ratio between the rods and the plates. A steep slope is expected if the plates are relatively small, and a gentle slope is expected if the plates are relatively large.

**Figure 3:**
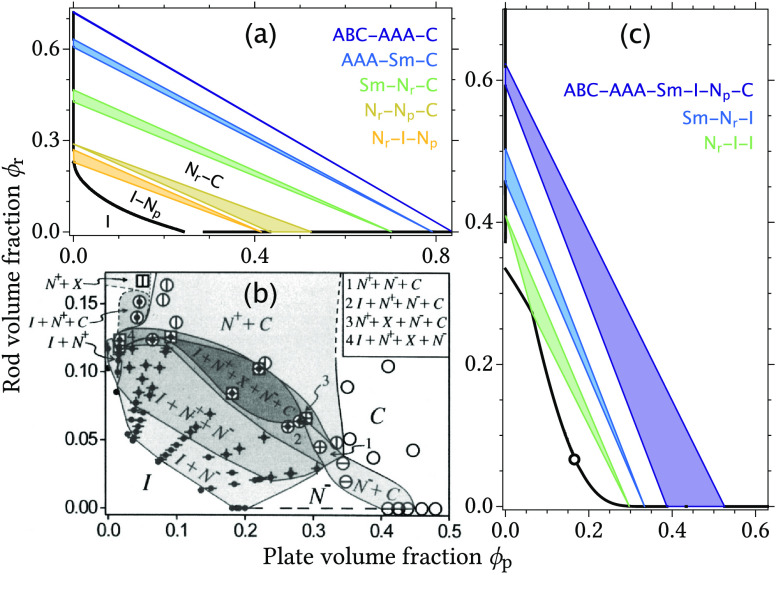
(a) Theoretically predicted phase diagram for a colloidal dispersion of hard rods and hard plates with a rod aspect ratio *L*_r_/*D*_r_ of 10, a plate aspect ratio *L*_p_/*D*_p_ of 1/15, and a size ratio *L*_r_/*D*_p_ of 4/3. N_r_ and N_p_ indicate nematic phases rich in rods and rich in plates, respectively. (b) Experimentally observed coexistence regions in a colloidal rod/plate mixture for similar parameters reproduced from Van der Kooij and Lekkerkerker.^24^ N^+^ and N^–^ indicate nematic phases rich in rods and rich in plates, respectively. (c) Theoretically predicted phase diagram containing a six-phase ABC–AAA–Sm–I–N_p_–C coexistence region. The aspect ratios are *L*_r_/*D*_r_ = 5.61 and *L*_p_/*D*_p_ = 0.12. The size ratio is *L*_r_/*D*_p_ = 1.92.

Eckert et al.^43^ used DFT to compute a phase diagram for a rod/plate mixture with the same parameters as in Figure 3a, but without the C, Sm, AAA, and ABC phases. Their predictions for the N_r_–I–N_p_ coexistence region are similar to our predictions; however, the stability regions of the homogeneous N_p_ and N_r_ phases are larger than the corresponding regions in Figure 3a. This is likely due to the general SPT approach used in this Letter for the work of insertion that overestimates the work needed to insert a particle in an ordered phase such as the nematic phase where rods or plates are orientationally aligned.

Figure 3b shows an experimentally measured phase diagram^3,24^ of a mixture of rod-like boehmite and plate-like gibbsite colloids with particle sizes that correspond roughly to the parameters in Figure 3a (within the experimental uncertainty). Qualitatively, the theoretical and experimental phase diagrams are very similar. At low concentrations, a transition from a homogeneous isotropic phase to an I–N_p_ coexistence region is found. At slightly higher concentrations, N_r_–I–N_p_, N_r_–N_p_–C, and N_r_–I–N_p_–C multiphase coexistence regions are found. The same three-phase coexistence regions are present in the theoretical predictions, which also show that the parameters are very close to the parameters for which the four-phase coexistence region is expected. At concentrations above the multiphase coexistence regions, N_r_–C coexistence is reported^3,24^ similar to our theoretical prediction. The experimental phase diagram also shows a region where a five-phase coexistence was observed with an additional phase denoted as “X”, which could not be unambiguously defined^3^ but somewhat resembles the smectic A phase considered in our theoretical framework.

The experimentally observed quintuple phase coexistence region was attributed to the polydispersity of the colloidal rods and plates because the classical Gibbs phase rule does not allow for coexistence among five different phases in a two-component mixture. However, as explained above, the generalized Gibbs phase rule (eq 3) does allow for this phenomenon. Size polydispersity, present in most colloidal mixtures, does affect the multiphase coexistence behavior, and it is expected that polydispersity smears out the theoretically predicted coexistence regions and enhances the probability of finding coexistence regions with multiple phases such as the observed quintuple phase coexistence region by Van der Kooij and Lekkerkerker.^3,24^

The generalized Gibbs phase rule predicts that even a sextuple phase coexistence region is theoretically possible in a mixture of colloidal rods and plates, because such mixtures have three additional parameters that affect the phase behavior : the aspect ratio of the rods, the aspect ratio of the plates, and the size ratio between rods and plates. With the presented theoretical framework, we indeed find a phase diagram containing a six-phase ABC–AAA–Sm–I–N_p_–C equilibrium for certain parameters as shown in Figure 3c. The additional free parameter with respect to rod/sphere mixtures means that mapping the phase behavior in a similar fashion as in Figure 1 is quite demanding as it adds a third dimension to the phase coexistence overview. The phase behavior of colloidal mixtures becomes even more extensive if particles with more than one shape parameter or with more than two components are considered.

The general theoretical framework outlined in this Letter was used to showcase the rich phase behavior of binary colloidal mixtures. The possibility of both colloidal components forming ordered phases allows for a wide variety of multiphase coexistence regions, including two quintuple phase coexistence regions for rod/sphere mixtures and even a sextuple phase coexistence region for rod/plate mixtures. The presented theory is of general interest for applications of colloidal mixtures and can be used for example to find size ratios and shape parameters for which the stability region of the homogeneous binary fluid phase is largest or for which parameters it is likely to find multiple different phases with similar thermodynamic properties. For accurate quantitative predictions of the phase boundaries, it should be noted that the accuracy of the equations of state used for the colloidal mixtures is crucial. Further verification of the free energy expressions used in this Letter with computer simulations is desirable; however, reports of such studies are scarce. For ordered phases of specific colloidal mixtures, a combination of cell theory and scaled particle theory^56,70^ can potentially be applied to improve the predictions for the work of insertion for the minor component. The presented theory can also be extended to multicomponent mixtures, which increases the complexity of the phase behavior by adding further dimensions to the phase diagrams. Furthermore, for future work, incorporation of mixed phases in which both components are ordered in the presented theoretical framework such as biaxial phases or binary crystals is desired. These colloidal phases have characteristics that are of interest for varying applications; however, developing a general method for obtaining approximate equations of state for phases of colloidal mixtures in which multiple components are ordered remains challenging.

## Supporting Information Available

Extended theory section, with section 1 giving a brief derivation of the free energy of a binary colloidal mixture as defined in eq 1, section 2 showing how to extend the equations to mixtures containing more than two components, section 3 showing the connection between eq 1 and the semi-grand canonical potential used in free volume theory, section 4 summarizing the free energy expressions used for one-component sphere, rod, and plate dispersions used as input for eq 1, section 5 outlining the general scaled particle theory method used for the determination of the work of insertion, and section 6 showing an overview of representative phase diagrams found in the different indicated regions of Figure 1 (PDF)
Transparent Peer Review report available (PDF)

## Notes

Mathematica notebooks used for the calculations in this Letter are available upon request.
