# Supplementary material for: Multiphase Coexistence in Binary Hard Colloidal Mixtures: Predictions from a Simple Algebraic Theory
Source: J Phys Chem Lett. 2022 Dec 29;14(1):199–206. doi: 10.1021/acs.jpclett.2c03138 (PMC9841575; doi:10.1021/acs.jpclett.2c03138)
Supplement: Supplementary file 1 — jz2c03138_si_001.pdf [file jz2c03138_si_001.pdf]

Supporting Information for "Multi-Phase Coexistence in Binary Hard Colloidal Mixtures: Predictions from a Simple Algebraic Theory"

J. Opdam,[†] V. F. D. Peters,[†,‡] H. H. Wensink,[¶] and R. Tuinier[*,†,§]

† *Laboratory of Physical Chemistry, Department of Chemical Engineering and Chemistry, & Institute for Complex Molecular Systems (ICMS), Eindhoven University of Technology, P.O. Box 513, 5600 MB, Eindhoven, The Netherlands*

‡*Department of Earth Sciences, Utrecht University, Princetonlaan 8a, 3584CB Utrecht, The Netherlands*

¶*Laboratoire de Physique des Solides, Université Paris-Saclay & CNRS, 91405 Orsay, France*

§*Corresponding author*

E-mail: r.tuinier@tue.nl

This supporting information provides an extended theory section and representative phase diagrams for rod/sphere mixtures with various size ratios and rod aspect ratios. Spherical particles have diameter D_s, rod-like particles are considered as spherocylinders with diameter D_r and length L_r, plate-like particles are considered as cylinders with diameter D_p and length L_p. Normalized quantities are defined as follows:

$$\begin{aligned} \widetilde{F} &= \frac{F\,v_1}{V\,k_\mathrm{B}T} \quad , \quad \widetilde{\mu} = \frac{\mu}{k_\mathrm{B}T} \quad , \quad \widetilde{\Pi} = \frac{\Pi\,v_1}{k_\mathrm{B}T} \quad , \\ \widetilde{W} &= \frac{W}{k_\mathrm{B}T} \quad , \quad \alpha_i = \frac{\langle V_{\mathrm{free},i}\rangle_0}{V} \quad , \quad \phi_i = \frac{N_i v_i}{V} \quad , \end{aligned} \tag{S1}$$

where $\langle V_{\mathrm{free},i}\rangle$ is the ensemble-averaged volume that is available for component i, k_B is the Boltzmann constant, T is the temperature and V the volume of the system.

Helmholtz free energy

The free energy $\widetilde{F}$ of an athermal mixture of two types of colloidal particles, labeled 1 and 2, is given by

$$\widetilde{F} = -\widetilde{\Pi} + \widetilde{\mu}_1\phi_1 + \frac{v_1}{v_2}\widetilde{\mu}_2\phi_2 \quad , \tag{S2}$$

with Π the osmotic pressure of the mixture, μ the chemical potential and ϕ the volume fraction. v_1 and v_2 are the volumes of particle 1 and particle 2 respectively. The tildes indicate that normalized quantities are used as defined by Eq. S1. The particle volume of component 1 is used here for normalization. The change in free energy can than be written down as

$$\mathrm{d}\widetilde{F} = \widetilde{\mu}_1\mathrm{d}\phi_1 + \frac{v_1}{v_2}\widetilde{\mu}_2\mathrm{d}\phi_2 \quad , \tag{S3}$$

where a constant volume is assumed and the Gibbs–Duhem equation for a binary mixture is applied:

$$\mathrm{d}\widetilde{\Pi} = \phi_1 \mathrm{d}\widetilde{\mu}_1 + \frac{v_1}{v_2}\phi_2 \mathrm{d}\widetilde{\mu}_2 \quad . \tag{S4}$$

Eq. S3 can be rewritten in terms of partial derivatives of the free energy:

$$\mathrm{d}\widetilde{F} = \left(\frac{\partial \widetilde{F}}{\partial \phi_1}\right)_{\phi_2} \mathrm{d}\phi_1 + \left(\frac{\partial \widetilde{F}}{\partial \phi_2}\right)_{\phi_1} \mathrm{d}\phi_2 \quad . \tag{S5}$$

The free energy of the binary mixture as a perturbation on the free energy of a one-component system can be found by integration of Eq. S5:

$$\widetilde{F} = \int_0^{\phi_1} \left(\frac{\partial \widetilde{F}}{\partial \phi_1'}\right)_{\phi_2=0} \mathrm{d}\phi_1' + \int_0^{\phi_2} \left(\frac{\partial \widetilde{F}}{\partial \phi_2'}\right)_{\phi_1} \mathrm{d}\phi_2' \quad , \tag{S6a}$$

$$\widetilde{F} = \widetilde{F}_{\phi_2=0} + \int_0^{\phi_2} \frac{v_1}{v_2}\widetilde{\mu}_2 \mathrm{d}\phi_2' \quad . \tag{S6b}$$

Finally, the free energy can be expressed in terms of the work $\widetilde{W}_2$ needed to insert an additional particle of component 2 into the binary mixture using Widom's insertion theory:[1]

$$\widetilde{F} = \widetilde{F}_{\phi_2=0} + \frac{v_1}{v_2}\left(\phi_2\widetilde{\mu}_2^0 + \phi_2 \ln \phi_2 - \phi_2 + \int_0^{\phi_2} \widetilde{W}_2 \mathrm{d}\phi_2'\right) \quad , \tag{S7}$$

where the chemical potential of component 2 is defined as

$$\widetilde{\mu}_2 = \widetilde{\mu}_2^0 + \ln \phi_2 + \widetilde{W}_2 \quad , \tag{S8}$$

with $\widetilde{\mu}_2^0 = \ln(\Lambda^3/v_2)$ and Λ the De Broglie wavelength. Equivalently, the free energy can also be described as a perturbation of the free energy of a system containing only component 2:

$$\widetilde{F} = \frac{v_1}{v_2}\widetilde{F}_{\phi_1=0} + \left(\phi_1\widetilde{\mu}_1^0 + \phi_1 \ln \phi_1 - \phi_1 + \int_0^{\phi_1} \widetilde{W}_1 \mathrm{d}\phi_1'\right) \quad . \tag{S9}$$

Free energy of multi-component colloidal mixtures

The free energy expression given by Eq. S7 can be extended to more than two components. For an athermal mixture of three colloidal particles the free energy is given by

$$\widetilde{F} = -\widetilde{\Pi} + \widetilde{\mu}_1\phi_1 + \frac{v_1}{v_2}\widetilde{\mu}_2\phi_2 + \frac{v_1}{v_3}\widetilde{\mu}_3\phi_3 \quad . \tag{S10}$$

The total derivative can then be written down as

$$\mathrm{d}\widetilde{F} = \left(\frac{\partial \widetilde{F}}{\partial \phi_1}\right)_{\phi_2,\phi_3} \mathrm{d}\phi_1 + \left(\frac{\partial \widetilde{F}}{\partial \phi_2}\right)_{\phi_1,\phi_3} \mathrm{d}\phi_2 + \left(\frac{\partial \widetilde{F}}{\partial \phi_3}\right)_{\phi_1,\phi_2} \mathrm{d}\phi_3 \quad . \tag{S11}$$

Following similar steps as in the previous section, the free energy as a function of the free energy of a system containing only component 1 and the work of insertion of particles of type 2 and 3 is obtained:

$$\widetilde{F} = \int_0^{\phi_1} \left(\frac{\partial \widetilde{F}}{\partial \phi_1'}\right)_{\phi_2=0,\phi_3=0} \mathrm{d}\phi_1' + \int_0^{\phi_2} \left(\frac{\partial \widetilde{F}}{\partial \phi_2'}\right)_{\phi_1,\phi_3=0} \mathrm{d}\phi_2' + \int_0^{\phi_3} \left(\frac{\partial \widetilde{F}}{\partial \phi_3'}\right)_{\phi_1,\phi_2} \mathrm{d}\phi_3' \quad , \tag{S12a}$$

$$\widetilde{F} = \widetilde{F}_{\phi_2=0,\phi_3=0} + \int_0^{\phi_2} \left(\widetilde{\mu}_2\right)_{\phi_3=0} \mathrm{d}\phi_2' + \int_0^{\phi_3} \widetilde{\mu}_3 \mathrm{d}\phi_3' \quad , \tag{S12b}$$

$$\widetilde{F} = \widetilde{F}_{\phi_2=0,\phi_3=0} + \sum_{i=2}^{3} \frac{v_1}{v_i}\phi_i \left(\widetilde{\mu}_i^0 + \ln\phi_i - 1\right) + \frac{v_1}{v_2}\int_0^{\phi_2} \left(\widetilde{W_2}\right)_{\phi_3=0} \mathrm{d}\phi_2' + \frac{v_1}{v_3}\int_0^{\phi_3} \widetilde{W_3} \mathrm{d}\phi_3' \quad , \tag{S12c}$$

with $\left(\widetilde{W_2}\right)_{\phi_3=0}$ the work of insertion for component 2 into a binary mixture of components 1 and 2 and $\widetilde{W_3}$ is the work of insertion for component 3 in a ternary mixture of components 1, 2 and 3. Generalizing Eq. S12c for a mixture of n particles or polydisperse particles is straight forward.

Connection with free volume theory

In FVT, originally developed for colloid/polymer mixtures by Lekkerkerker *et al.*,[2,3] one component is described canonically with a fixed number of particles and the second component, the "depletant", is described with a grand canonical ensemble where the number of depletants in the system is not known *a priori*, but determined through an equilibrium with a hypothetical depletant reservoir with a fixed chemical potential of the depletants. The thermodynamic properties of the mixture are described by the semi-grand potential which is defined as:

$$\widetilde{\Omega} = \widetilde{F} - \frac{v_1}{v_2}\phi_2\widetilde{\mu}_2 \quad . \tag{S13}$$

For a binary mixture of colloidal particles, this expression can be approximated by:[4]

$$\widetilde{\Omega} = \widetilde{F}_{\phi_2=0} - \frac{v_1}{v_2}\int_0^{\phi_2^{\mathrm{R}}} \frac{\alpha_2}{\alpha_2^{\mathrm{R}}}\left(\frac{\partial \widetilde{\Pi}^{\mathrm{R}}}{\partial \phi_2^{\mathrm{R}\prime}}\right)\mathrm{d}\phi_2^{\mathrm{R}\prime} \quad , \tag{S14}$$

where the superscript "R" denotes the reservoir and α_2 describes the free volume available for component 2.

To make a connection between the semi-grand potential of Eq. S14 and the free energy expression of Eq. S7 (which is Eq. 1 in the main Letter), we start by combining Eq. S7 with Eq. S13:

$$\widetilde{\Omega} = \widetilde{F}_{\phi_2=0} + \frac{v_1}{v_2}\left(\phi_2\widetilde{\mu}_2^0 + \phi_2\ln\phi_2 - \phi_2 + \int_0^{\phi_2}\widetilde{W}_2\mathrm{d}\phi_2'\right) - \frac{v_1}{v_2}\phi_2\mu_2 \quad . \tag{S15}$$

This expression can be simplified using Eq. S8:

$$\widetilde{\Omega} = \widetilde{F}_{\phi_2=0} + \frac{v_1}{v_2}\left(-\phi_2 + \int_0^{\phi_2}\widetilde{W}_2\mathrm{d}\phi_2' - \phi_2\widetilde{W}_2\right) \quad . \tag{S16}$$

Rewriting the first and last term within the brackets as an integral of its derivative results in:

$$\widetilde{\Omega} = \widetilde{F}_{\phi_2=0} - \frac{v_1}{v_2}\left[-\int_0^{\phi_2} \widetilde{W}_2 \mathrm{d}\phi_2' + \int_0^{\phi_2}\left(1 + \phi_2' \frac{\partial \widetilde{W}_2}{\partial \phi_2'} + \widetilde{W}_2\right)\mathrm{d}\phi_2'\right] \quad , \tag{S17a}$$

$$\widetilde{\Omega} = \widetilde{F}_{\phi_2=0} - \frac{v_1}{v_2}\int_0^{\phi_2}\left(1 + \phi_2' \frac{\partial \widetilde{W}_2}{\partial \phi_2'}\right)\mathrm{d}\phi_2' \quad . \tag{S17b}$$

Next, we again make use of Eq. S8 to rewrite the obtained expression:

$$\widetilde{\Omega} = \widetilde{F}_{\phi_2=0} - \frac{v_1}{v_2}\int_0^{\phi_2} \phi_2' \left(\frac{\partial \widetilde{\mu}_2}{\partial \phi_2'}\right)\mathrm{d}\phi_2' \quad . \tag{S18}$$

The volume fraction of component 2 can be described as a function of the volume fraction of component 2 in the reservoir through equilibrium conditions resulting in:[4]

$$\frac{\phi_2}{\phi_2^{\mathrm{R}}} = \frac{\alpha_2}{\alpha_2^{\mathrm{R}}} \quad , \tag{S19}$$

where it is assumed that component 2 does not exhibit translational or orientational order and only interacts through excluded volume interactions with itself and component 1. Combining Eqs. S18 and S19 gives:

$$\widetilde{\Omega} = \widetilde{F}_{\phi_2=0} - \frac{v_1}{v_2}\int_0^{\phi_2^{\mathrm{R}}} \phi_2^{\mathrm{R}'} \frac{\alpha_2}{\alpha_2^{\mathrm{R}}} \left(\frac{\partial \widetilde{\mu}_2}{\partial \phi_2^{\mathrm{R}'}}\right)\mathrm{d}\phi_2^{\mathrm{R}'} \quad , \tag{S20}$$

which finally yields the FVT expression of Eq. S14 by applying the Gibbs–Duhem equation.

Free energy expressions for one-component phases

The free energy expressions used for an effective one-component system of spheres, rods or plates used in the main letter are outlined below. The expressions are stated for the purpose of reproducibility, for details the reader is referred to references[5] and[6] for rods and plates, respectively.

Spheres

The Helmholtz free energy $\widetilde{F}_0$ of the pure hard sphere fluid phase is given by

$$\widetilde{F}_{\rm s}^{\rm F} = \phi_{\rm s}\left[\ln\left(\phi_{\rm s}\Lambda^3/v_{\rm s}\right) - 1\right] + \frac{4\phi_{\rm s}^2 - 3\phi_{\rm s}^3}{(1-\phi_{\rm s})^2} \quad , \tag{S21}$$

where the first term on the right-hand side is the ideal contribution and the second term originates from the Carnahan–Starling equation of state.[7] For the hard sphere solid, a face-centered-cubic (FCC) crystal, the result from Lennard-Jones and Devonshire cell theory[8] is used:

$$\widetilde{F}_{\rm s}^{\rm FCC} = \phi_{\rm s}\ln\left(\Lambda^3/v_{\rm s}\right) + \phi_{\rm s}\ln\left(\frac{27}{8\phi_{\rm cp}^3}\right) + 3\phi_{\rm s}\ln\left(\frac{\phi_{\rm s}}{1-\phi_{\rm s}/\phi_{\rm cp}}\right) \quad , \tag{S22}$$

with $\phi_{\rm cp}$ the volume fraction of a close-packed FCC crystal ($\phi_{\rm cp} = \pi/(3\sqrt{2}) \approx 0.74$).

Rods

SPT is used for the free energy of the fluid and nematic phases of rods:[9]

$$\frac{\widetilde{F}_{\rm r}^{\rm F}}{\phi_{\rm r}} = \widetilde{F}_{\rm r,ideal} - \ln(1-\phi_{\rm r}) + \frac{3\gamma(1+\gamma)}{3\gamma-1}\frac{\phi_{\rm r}}{1-\phi_{\rm r}} + \frac{6\gamma^3}{(1-3\gamma)^2}\frac{\phi_{\rm r}^2}{(1-\phi_{\rm r})^2} \quad , \tag{S23}$$

$$\frac{\widetilde{F}_{\rm r}^{\rm N}}{\phi_{\rm r}} = \widetilde{F}_{\rm r,ideal} - \ln(1-\phi_{\rm r}) + \left[3 + \frac{4}{\sqrt{\pi\kappa_{\rm r,N}}}\frac{3(\gamma-1)^2}{3\gamma-1}\right]\frac{\phi_{\rm r}}{1-\phi_{\rm r}} + \frac{6\gamma}{(1-3\gamma)^2}\left[\frac{4(\gamma-1)^2}{\sqrt{\pi\kappa_{\rm r,N}}} + 2\gamma - 1\right]\frac{\phi_{\rm r}^2}{(1-\phi_{\rm r})^2} + \ln\kappa_{\rm r,N} - 1.139 \quad , \tag{S24}$$

with γ related to the rod aspect ratio $\gamma = 1 + L_{\rm r}/D_{\rm r}$ and

$$\kappa_{\rm r,N} = \frac{9(\gamma-1)^4}{4\pi(3\gamma-1)^2}\left[\frac{4\phi_{\rm r}}{1-\phi_{\rm r}} + \frac{8\gamma}{3\gamma-1}\frac{\phi_{\rm r}^2}{(1-\phi_{\rm r})^2}\right]^2 \quad . \tag{S25}$$

The ideal free energy $\widetilde{F}_{\rm r,ideal}$ is given by $\widetilde{F}_{\rm r,ideal} = \ln(\phi_{\rm r}\Lambda^3/v_{\rm r}) - 1$. The free energy of the

Table 1: Values of the constants c_i used in Eq. S27

c_0	12	c_1	-385	c_2	-4980	c_3	75048	c_4	-288904
c_5	534956	c_6	-553098	c_7	328296	c_8	-105024	c_9	14080

smectic phase is derived through cell theory and the resulting expression is as follows:

$$\frac{\widetilde{F}_{\mathrm{r}}^{\mathrm{Sm}}}{\phi_{\mathrm{r}}} = \widetilde{F}_{\mathrm{r,ideal}} + 2\ln\kappa_{\mathrm{r,Sm}} - 2 - \ln\left(1 - \phi_{\mathrm{r,2D}}\widetilde{D}_{\mathrm{r,eff}}^{2}\right) + \frac{\phi_{\mathrm{r,2D}}\widetilde{D}_{\mathrm{r,eff}}^{2}}{1 - \phi_{\mathrm{r,2D}}\widetilde{D}_{\mathrm{r,eff}}^{2}} - \ln\left(1 - \frac{\gamma}{\widetilde{\Delta}_{\mathrm{r},\perp}}\right) \quad , \tag{S26}$$

where the parameters $\kappa_{\mathrm{r,Sm}}$, $\widetilde{D}_{\mathrm{r,eff}}$, $\phi_{\mathrm{r,2D}}$ and $\widetilde{\Delta}_{\mathrm{r},\perp}$ are defined as

$$\kappa_{\mathrm{r,Sm}} = 0.82\phi_{\mathrm{r}}(\gamma-1)\frac{6\phi_{\mathrm{r,2D}} - 5\phi_{\mathrm{r,2D}}^{2}}{\left(1-\phi_{\mathrm{r,2D}}\right)^{2}} \quad , \quad \widetilde{D}_{\mathrm{r,eff}} = 1 + \frac{0.82\phi_{\mathrm{r}}(\gamma-1)}{\kappa_{\mathrm{r,Sm}}} \quad , \quad \phi_{\mathrm{r,2D}} = \frac{\zeta\widetilde{\Delta}_{\mathrm{r},\perp}}{\gamma} \quad ,$$
$$\widetilde{\Delta}_{\mathrm{r},\perp} = \gamma\left[1 + \frac{(6-5\zeta)(1-\zeta)^{2}(1-27\zeta+41\zeta^{2}-16\zeta^{3})^{2}}{\sum_{i=0}^{9} c_i\zeta^{i}}\right] \quad , \quad \zeta = \frac{3\gamma}{3\gamma-1}\phi_r \quad . \tag{S27}$$

The values of the constants c_i in Eq. S27 are listed in Table 1. The free energy of the AAA and ABC phases are also derived through cell theory and have a similar free energy expression:

$$\frac{\widetilde{F}_{\mathrm{r}}^{\mathrm{AAA,ABC}}}{\phi_{\mathrm{r}}} = 2\ln\kappa_{\mathrm{r},i} - 2 + \ln\left(\frac{\Lambda^{3}}{v_{\mathrm{r}}}\right) + \ln\left(\phi_{\mathrm{r}}x_{\mathrm{r},i}\right) - \ln\left(\widetilde{\Delta}_{\mathrm{r},\parallel,i} - \widetilde{D}_{\mathrm{r,eff},i}\right)^{2} - \ln\left(\frac{x_{\mathrm{r},i}}{\widetilde{\Delta}_{\mathrm{r},\parallel,i}^{2}} - 1\right) \quad , \tag{S28}$$

with i denoting either the AAA or ABC phase and the parameter $\widetilde{\Delta}_{\mathrm{r},\parallel,i}$ defined as

$$\widetilde{\Delta}_{\mathrm{r},\parallel,i} = \frac{6^{1/3}x_{\mathrm{r},i} + \left[9x_{\mathrm{r},i} + x_{\mathrm{r},i}\sqrt{3(27-2x_{\mathrm{r},i})}\right]^{2/3}}{6^{2/3}x_{\mathrm{r},i}\left[9x_{\mathrm{r},i} + x_{\mathrm{r},i}\sqrt{3(27-2x_{\mathrm{r},i})}\right]^{1/3}} \quad , \tag{S29}$$

and the parameters $x_{\mathrm{r},i}$, $\kappa_{\mathrm{r},i}$ and $\widetilde{D}_{\mathrm{r,eff},i}$ in Eqs. S28 and S29 are different for the AAA and for the ABC phase. For the AAA phase, the parameters are defined by the following expressions:

$$x_{\mathrm{r,AAA}} = \frac{\pi(3\gamma-1)}{6\phi_{\mathrm{r}}\sqrt{3}\gamma} \quad , \quad \kappa_{\mathrm{r,AAA}} = \frac{0.9\phi_{\mathrm{r}}(\gamma-1)}{\widetilde{\Delta}_{\mathrm{r},\parallel,\mathrm{AAA}}-1} \quad , \quad \widetilde{D}_{\mathrm{r,eff,AAA}} = 1 + 0.225\phi_{\mathrm{r}}(\gamma-1)\frac{2}{\kappa_{\mathrm{r,AAA}}} \quad , \tag{S30}$$

and for the ABC phase:

$$x_{\mathrm{r,ABC}} = \frac{\pi(3\gamma-1)}{6\phi_{\mathrm{r}}\left[\sqrt{3}(\gamma-1)+1.16\sqrt{2}\right]} \quad , \quad \kappa_{\mathrm{r,ABC}} = \frac{0.956\phi_{\mathrm{r}}(\gamma-1)}{\widetilde{\Delta}_{\mathrm{r},\parallel,\mathrm{ABC}}-1} \quad ,$$
$$\widetilde{D}_{\mathrm{r,eff,ABC}} = 1 + 0.239\phi_{\mathrm{r}}(\gamma-1)\frac{2}{\kappa_{\mathrm{r,ABC}}} \quad . \tag{S31}$$

Plates

The free energy expressions for the fluid and nematic phases of hard plates are derived using Parsons–Lee theory, resulting in:

$$\frac{\widetilde{F}^{\mathrm{F}}_{\mathrm{p}}}{\phi_{\mathrm{p}}} = \widetilde{F}_{\mathrm{p,ideal}} + \frac{4\phi_{\mathrm{p}}-3\phi_{\mathrm{p}}^2}{2\pi\varGamma(1-\phi_{\mathrm{p}})^2}\left(\frac{\pi^2}{8}+\frac{3\pi\varGamma}{4}+\frac{\pi^2\varGamma}{4}+\frac{\pi\varGamma^2}{2}\right) \quad , \tag{S32}$$

$$\frac{\widetilde{F}^{\mathrm{N}}_{\mathrm{p}}}{\phi_{\mathrm{p}}} = \widetilde{F}_{\mathrm{p,ideal}} + \ln\kappa_{\mathrm{p,N}} - 1 + \frac{4\phi_{\mathrm{p}}-3\phi_{\mathrm{p}}^2}{2\pi\varGamma(1-\phi_{\mathrm{p}})^2}\left(\frac{\pi^{3/2}}{2\sqrt{\kappa_{\mathrm{p,N}}}}+2\pi\varGamma\right) \quad , \tag{S33}$$

with the plate aspect ratio $\Gamma = L_{\mathrm{p}}/D_{\mathrm{p}}$ and

$$\kappa_{\mathrm{p,N}} = \frac{\pi\left(4\phi_{\mathrm{p}}-3\phi_{\mathrm{p}}^2\right)^2}{64\varGamma^2(1-\phi_{\mathrm{p}})^4} \quad , \tag{S34}$$

and the ideal free energy $\widetilde{F}_{\mathrm{p,ideal}}$ given by $\widetilde{F}_{\mathrm{p,ideal}} = \ln\left(\phi_{\mathrm{p}}\varLambda^3/v_{\mathrm{p}}\right) - 1$. The free energy of the columnar phase is obtained through LJD cell theory:

$$\frac{\widetilde{F}_{\mathrm{p}}^{\mathrm{C}}}{\phi_{\mathrm{p}}} = \widetilde{F}_{\mathrm{p,ideal}} + 2\ln\left[\frac{3x_{\mathrm{p}}\widetilde{\Delta}_{\mathrm{p}}^{2}}{2\Gamma\left(1 - x_{\mathrm{p}}\widetilde{\Delta}_{\mathrm{p}}\right)^{2}}\right] - 2 - \ln\left(\frac{1 - x_{\mathrm{p}}\widetilde{\Delta}_{\mathrm{p}}^{2}}{3}\right) - 2\ln\left(1 - \frac{1}{\widetilde{\Delta}_{\mathrm{p}}}\right) - \ln 4 \quad , \qquad \text{(S35)}$$

with the parameters x_{p} and $\widetilde{\Delta}_{\mathrm{p}}$ given by

$$x_{\mathrm{p}} = \frac{2\sqrt{3}\phi_{\mathrm{p}}}{\pi} \quad , \quad \widetilde{\Delta}_{\mathrm{p}} = \frac{2^{1/3}\kappa_{\mathrm{p,C}}^{2/3} - 4x_{\mathrm{p}}3^{1/3}}{6^{2/3}x_{\mathrm{p}}\kappa_{\mathrm{p,C}}^{1/3}} \quad , \quad \kappa_{\mathrm{p,C}} = 27x_{\mathrm{p}}^{2} + \sqrt{3x_{\mathrm{p}}^{3}(243x_{\mathrm{p}} + 32)} \quad . \qquad \text{(S36)}$$

Scaled particle theory

The general SPT expression for the work of insertion for a particle in a binary mixture of components 1 and 2 is derived below. In SPT, an expression is obtained by considering the insertion of a particle with a scaled size, which size and shape are described by λx, νy and σz. Here, x, y, z are parameters that define the size and shape of the particle, for example radius or length, and λ, ν and σ are scaling parameters. The work of insertion $\widetilde{W}_{2\mathrm{s}}$ for a scaled particle of component 2 in a binary colloidal mixture of components 1 and 2 can be written down as a Taylor expansion connecting the limits of inserting a very small and a very large particle:

$$\widetilde{W}_{2\mathrm{s}}\left(\lambda, \nu, \sigma\right) = \widetilde{W}_{2,\mathrm{T}} + \widetilde{W}_{2\mathrm{s}}\left(\lambda >> 1, \nu >> 1, \sigma >> 1\right) \quad , \qquad \text{(S37)}$$

with

$$\widetilde{W}_{2,\mathrm{T}} = \sum_{i=0}^{\mathcal{D}_x}\sum_{j=0}^{\mathcal{D}_y}\sum_{k=0}^{\mathcal{D}_z}{}_{i+j+k<3} \frac{1}{i!j!k!}\left[\frac{\partial^{i+j+k}}{\partial\lambda^{i}\partial\nu^{j}\partial\sigma^{k}}\widetilde{W}_{2\mathrm{s}}\left(\lambda \to 0\, \nu \to 0\, \sigma \to 0\right)\right]_{\lambda,\nu,\sigma=0} \lambda^{i}\nu^{j}\sigma^{k} \quad . \qquad \text{(S38)}$$

Here, $\mathcal{D}_x$, $\mathcal{D}_y$ and $\mathcal{D}_z$ denote the power-scaling of the particle volume as a function of the

parameters x, y, z. The condition that $i + j + k < 3$ implies that higher order terms are accounted for by the last term on the right-hand side of Eq. S37. For spherical particles, $x = D_\mathrm{s}$ and $\mathcal{D}_x = 3$, hence $y = z = 0$ and $\mathcal{D}_y = \mathcal{D}_z = 0$. For rod-like particles and disks $x = D$ and $\mathcal{D}_x = 2$, $y = L$ and $\mathcal{D}_y = 1$, $z = 0$ and $\mathcal{D}_z = 0$. The work of insertion for a particle with the size of interest is obtained with

$$\widetilde{W}_2 = \widetilde{W}_{2\mathrm{s}}\left(\lambda = 1, \nu = 1, \sigma = 1\right) \quad . \tag{S39}$$

The last term on the right-hand side of equation S37 corresponds to the limit of large particle insertion and describes the work needed to create a cavity in the system with the size of the scaled insertion particle. This is directly related to the osmotic pressure in the binary mixture:

$$\widetilde{W}_{2\mathrm{s}}\left(\lambda >> 1, \nu >> 1, \sigma >> 1\right) = \frac{v_{2\mathrm{s}}}{v_1}\widetilde{\Pi} \quad . \tag{S40}$$

An expression for the limit of small particle insertion is obtained through the connection between $\widetilde{W}$ and the free volume fraction α:

$$\widetilde{W}_{2\mathrm{s}}\left(\lambda \to 0, \nu \to 0, \sigma \to 0\right) = -\ln \alpha_{2\mathrm{s}} = -\ln\left(1 - \phi_1 \frac{v_\mathrm{excl}^{1,2\mathrm{s}}}{v_1} - \phi_2 \frac{v_\mathrm{excl}^{2,2\mathrm{s}}}{v_2}\right) \quad , \tag{S41}$$

with $v_\mathrm{excl}^{1,2\mathrm{s}}$ and $v_\mathrm{excl}^{2,2\mathrm{s}}$ the excluded volumes of the inserted scaled particle 2s with respect to components 1 and 2, averaged over all orientations. Here it is assumed that there is no overlap between the excluded volume regions of different particles which is reasonable in the limit of small particle insertion. The orientationally averaged excluded volumes can be written in terms of the volume v, the surface area A and the integrated mean curvature c of the particles 1, 2 and 2s:[10]

Table 2: Volume v_i, surface area A_i and integrated mean curvature c_i of spheres, spherocylinders and cylinders (i = s, r and p, respectively). Expressions for the scaled particles are obtained by replacing R, D and L with λR, λD and νL, respectively.

i	v_i	A_i	c_i
s	$\frac{\pi}{6}D_\mathrm{s}^3$	πD_s^2	$\frac{D_\mathrm{s}}{2}$
r	$\frac{\pi}{6}D_\mathrm{r}^3 + \frac{\pi}{4}D_\mathrm{r}^2 L_\mathrm{r}$	$\pi D_\mathrm{r}^2 + \pi D_\mathrm{r} L_\mathrm{r}$	$\frac{L_\mathrm{r}}{4} + \frac{D_\mathrm{r}}{2}$
p	$\frac{\pi}{4}D_\mathrm{p}^2 L_\mathrm{p}$	$\frac{\pi}{2}D_\mathrm{p}^2 + \pi D_\mathrm{p} L_\mathrm{p}$	$\frac{\pi}{8}D_\mathrm{p} + \frac{L_\mathrm{p}}{4}$

$$v_\mathrm{excl}^{1,2\mathrm{s}} = v_1 + v_{2\mathrm{s}} + c_{2\mathrm{s}}A_1 + c_1 A_{2\mathrm{s}} \quad , \tag{S42}$$

$$v_\mathrm{excl}^{2,2\mathrm{s}} = v_2 + v_{2\mathrm{s}} + c_{2\mathrm{s}}A_2 + c_2 A_{2\mathrm{s}} \quad . \tag{S43}$$

The geometric properties of spheres, spherocylindrical rods and cylindrical are shown in Table 2.

The only thing left to calculate the work of insertion $\widetilde{W}_2$ is the osmotic pressure of the binary mixture $\widetilde{\Pi}$. The osmotic pressure can be obtained within the SPT treatment by writing the chemical potential of components 1 and 2 as a function of the work of insertion. Combining Eq. S8 with Eqs. S37–S40 and setting the scaling parameters equal to one gives the chemical potential of component 2 as a function of the osmotic pressure:

$$\widetilde{\mu}_2 = \widetilde{\mu}_2^0 + \ln\phi_2 + \widetilde{W}_{2,\mathrm{T}}\left(\lambda = 1, \nu = 1, \sigma = 1\right) + \frac{v_2}{v_1}\widetilde{\Pi} \quad , \tag{S44}$$

with $\widetilde{W}_{2,\mathrm{T}}$ defined by Eq. S38. Following the same approach as outlined above, but considering the insertion of a scaled particle of component 1 gives the chemical potential of component 1 as a function of the osmotic pressure:

$$\widetilde{\mu}_1 = \widetilde{\mu}_1^0 + \ln\phi_1 + \widetilde{W}_{1,\mathrm{T}}\left(\lambda = 1, \nu = 1, \sigma = 1\right) + \widetilde{\Pi} \quad . \tag{S45}$$

Combining Eqs. S44 and S45 with the Gibbs–Duhem equation

$$\left(\frac{\partial \widetilde{\Pi}}{\partial \phi}\right)_{f1} = \phi_1 \left(\frac{\partial \widetilde{\mu}_1}{\partial \phi}\right)_{f1} + \frac{v_1}{v_2}\phi_2 \left(\frac{\partial \widetilde{\mu}_2}{\partial \phi}\right)_{f1} \quad , \tag{S46}$$

yields a set of equations which can be solved to obtain an expression for the osmotic pressure. Here, $\phi = \phi_1 + \phi_2$ is the total volume fraction and $f_1 = \phi_1/\phi$ gives the fraction of particles that corresponds to component 1. This finally gives for the osmotic pressure:

$$\widetilde{\Pi} = \int_0^{\phi} \frac{\phi' f_1 \left[\frac{1}{\phi'} + \left(\frac{\partial \widetilde{W}_{1,\mathrm{T}}}{\partial \phi'}\right)_{f_1}\right] + \frac{v_1}{v_2}\phi'(1-f_1)\left[\frac{1}{\phi'} + \left(\frac{\partial \widetilde{W}_{2,\mathrm{T}}}{\partial \phi'}\right)_{f_1}\right]}{1-\phi'} \mathrm{d}\phi' \quad , \tag{S47}$$

where the volume fractions ϕ_1 and ϕ_2 in the Taylor expansions $\widetilde{W}_{1,\mathrm{T}}$ and $\widetilde{W}_{2,\mathrm{T}}$ are rewritten as a function of ϕ and f_1. After solving the integral of Eq. S47, $\widetilde{\Pi}$ can be simply rewritten in terms of ϕ_1 and ϕ_2. Eq. S47 provides the SPT expression for the osmotic pressure in a binary mixture, which is used in this chapter to obtain an analytical expression for $\widetilde{W}$. This keeps the work of insertion fully consistent with SPT, however, if a more accurate expression for the osmotic pressure of the binary mixture is available this can also be used in Eq. S40 instead of the SPT expression. Moreover, translational or orientational ordering of components affects the free volume available for the particles which is not accounted for in the SPT treatment. For specific phases, alternative but less general approaches[4,6,11] might be possible to obtain more accurate expressions for the work of insertion.

Representative phase diagrams for rod/sphere mixtures with varying size ratio and rod aspect ratio

Fig. S1 shows representative phase diagrams corresponding to each indicated region in the coexistence overview of Fig. 1a in the main text.

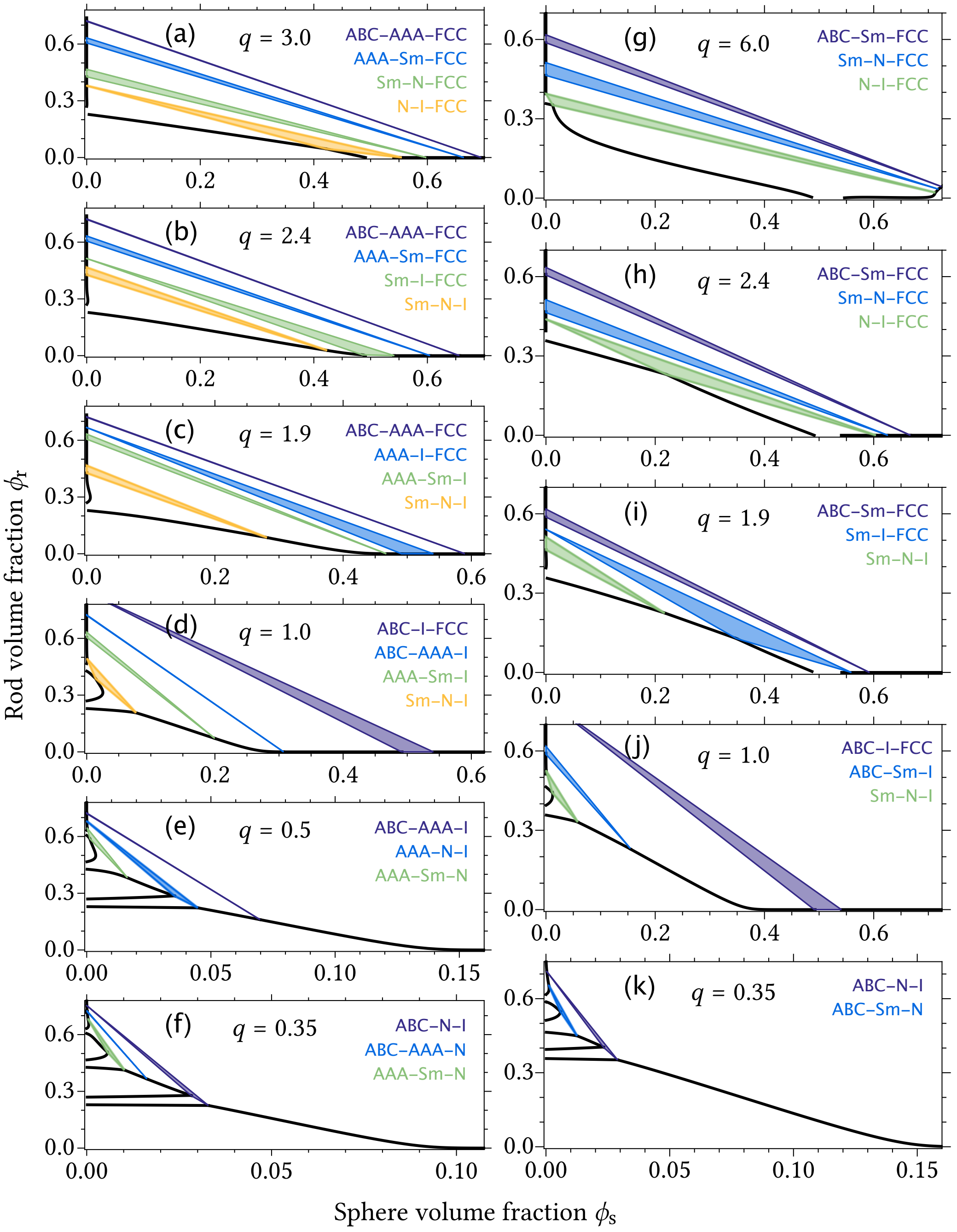

Figure S1: Representative phase diagrams of hard rod/sphere mixtures. Panels (a)–(f) correspond to regions I–VI and panels (h)–(k) correspond to regions VII–X indicated in Fig. 1a in the main text. Panel (g) corresponds to region VII, but with a larger size ratio than the range shown in Fig. 1a. Size ratios $q = D_s/D_r$ are indicated in the phase diagrams and the rod aspect ratio L_r/D_r equals 10 for panels (a)–(f) and 5 for panels (g)–(k). The type of phases for the multi-phase coexistence regions are indicated in the phase diagrams and the phases corresponding to the one-phase and two-phase regions follow accordingly. Equilibria containing the FCC phase are omitted for $q < 1$.

References

(1) Widom, B. Some topics in the theory of fluids. *J. Chem. Phys.* **1963**, *39*, 2808–2812.

(2) Lekkerkerker, H. N. W. Osmotic equilibrium treatment of the phase separation in colloidal dispersions containing non-adsorbing polymer molecules. *Colloids and Surfaces* **1990**, *51*, 419–426.

(3) Lekkerkerker, H. N. W.; Poon, W. C.-K.; Pusey, P. N.; Stroobants, A.; Warren, P. B. Phase behaviour of colloid + polymer mixtures. *Europhys. Lett.* **1992**, *20*, 559–564.

(4) Opdam, J.; Schelling, M. P. M.; Tuinier, R. Phase behaviour of binary hard-sphere mixtures. *J. Chem. Phys.* **2021**, *154*, 074902:1–11.

(5) Peters, V. F. D.; Vis, M.; Wensink, H. H.; Tuinier, R. Algebraic equations of state for the liquid crystalline phase behavior of hard rods. *Phys. Rev. E* **2020**, *101*, 062707:1–11.

(6) González García, Á.; Tuinier, R.; de With, G.; Cuetos, A. Directional-dependent pockets drive columnar-columnar coexistence. *Soft Matter* **2020**, *16*, 6720–6724.

(7) Carnahan, N. F.; Starling, K. E. Equation of state for nonattracting rigid spheres. *J. Chem. Phys.* **1969**, *51*, 635–636.

(8) Lennard-Jones, J. E.; Devonshire, A. F. Critical phenomena in gases-I. *Proc. R. Soc. A* **1937**, *163*, 53–70.

(9) Cotter, M. A. Hard-rod fluid: Scaled particle theory revisited. *Phys. Rev. A* **1974**, *10*, 625–636.

(10) Oversteegen, S. M.; Roth, R. General methods for free-volume theory. *J. Chem. Phys.* **2005**, *122*, 214502.

(11) Koda, T.; Ikeda, S. Test of the scaled particle theory for aligned hard spherocylinders using Monte Carlo simulation. *J. Chem. Phys.* **2002**, *116*, 5825–5830.
