# Supplementary material for: Multiphase Coexistence in Binary Hard Colloidal Mixtures: Predictions from a Simple Algebraic Theory
Source: J Phys Chem Lett. 2022 Dec 29;14(1):199–206. doi: 10.1021/acs.jpclett.2c03138 (PMC9841575; doi:10.1021/acs.jpclett.2c03138)
Supplement: Supplementary file 2 — jz2c03138_si_002.pdf [file jz2c03138_si_002.pdf]

jz-2022-03138x.R1

Name: Peer Review Information for "Multi-Phase Coexistence in Binary Hard Colloidal Mixtures: Predictions From a Simple Algebraic Theory"

First Round of Reviewer Comments

Reviewer: 1

Comments to the Author
The authors present in this work a proposal to calculate the free energy in multicomponent fluids. This theory incorporates the possibility that one of the components (and only one) is arranged in structured phases, with positional and/or positional order, while the other component behaves as a fluid. The authors use this theory to predict the phase diagram in binary mixtures of rod-shaped (spherocylinder) and spherical particles and disk-shaped (cylinder) and spherical particles. Both mixtures are studied over a wide range of parameters. The obtained phase diagrams show regions with coexistence of various phase numbers. These coexistences are rationalized by using the so-called generalized phase Gibbs rule.
This is a serious, deep and interesting work. It is well written, and the results are clearly presented. I believe that the theoretical approach can be very interesting for the study of a large number of systems. The results will undoubtedly be very interesting for a better understanding of the mixing of colloidal systems.
However, I have serious doubts about the discussion of the results. I think this discussion needs further reflection, and probably some extra work. My comments are as follows:
1º.- There is one aspect in the wording of the article that leads to confusion. In the introductory part of the article it is stated that
" However, these studies do not include the formation of positionally ordered phases of both components simultaneously. In this study, we allow both components to adopt orientational and positional ordered states and we showcase a wide range of multi-phase coexistences that can be reconfigured by tuning the principal size ratios of the mixture."

But further on, below equation 1 it is clarified that
"It is important to note that the Widom expression66 is used for the chemical potential of component 2, which means that component 2 is assumed to behave as a fluid phase with no orientational or translational order. This means that, using this approach, it is not possible to account for binary crystals or biaxial phases where both components are ordered."
I understand this to mean that it is not possible to calculate the free energy of phases in which both components are ordered. This is a limitation of the theory, which does not introduce the possibility of highly ordered phases that may be the most stable at high packing. This limitation makes the theory not valid in all cases, even though it maintains an important range of applicability.
But this sentence is contradictory to the previous sentence, which gives the theory a range of validity that the authors themselves admit it does not have. In fact, theories such as those

disqualified by the authors in the previous paragraph are also capable of predicting situations where one of the components is ordered, and the other is a fluid.
This aspect should be clarified by the authors, clarifying the range of applicability of their theory by indicating for which type of phases the theory can be applied. Here it is also important for the authors to point out the advantages of their theory over older theories. I think the authors need to spend some time justifying why their proposal is something more than "just another theory" (if it is).

2.-
One of the main limitations of the article is that the authors present little evidence of the accuracy of their theory. A qualitative comparison is made with the experimental phase diagram published by Van de Kooij and Lekkerkerkerker. As the authors point out, these experimental data are obtained from a system with a certain level of polydispersity, which makes a direct comparison doubtful. In contrast, no comparison with simulation results is made or discussed.

It is true that it can be difficult to find simulation results with complete phase diagrams as calculated by the authors. But there are published in the literature phase diagrams with some of the transitions indicated, especially at low packing.
Moreover, from the theory it is possible to obtain, in addition to the phase diagram, the equation of state in each of the phases found. The information on these equations of state for the systems studied in many of the phases is available in the literature, and if necessary can be calculated with not very complicate Monte Carlo simulations. It would be interesting for the authors to compare their theoretical predictions with these EOS obtained by simulation, and also to indicate whether this comparison between simulation and theory is better than in the case of other theories. I think it is something accessible, and it would give information on the range of validity of the proposed theory, and the improvement over previous works.

3.- The authors propose a modification to such a classical result as the Gibbs phase law. The truth is that I am not entirely comfortable with this proposal, with the so-called generalised Gibbs phase law.
This modification to the phase law was originally proposed by Vega and Monson, and then used (and analysed) by the authors in earlier publications. With these bibliographical precedents, and without having solid arguments to prove its invalidity, I understand that it is not my role as a reviewer to point out my doubts as a reason for not publishing the article. On the contrary, although I may find it shocking, since there are simulation results that seem to support the authors' interpretation,I believe that the possible controversy is a reason for publication, so that the community can discuss and pronounce itself on the subject. But I do not want to fail to point out here my doubts.
The main one is that I understand that the extension of Gibbs' law proposed by the authors would only make sense if the degrees of freedom associated with the dimensions of the particles were not frozen, if they could change. And this is not the case, since every system under study has fixed particle dimensions. I mean, although the study is extended for different

sizes of spheres, rods and disks, each phase diagram is calculated for fixed values of these dimensions.
In Vega and Moson's article, this rule is used to explain the appearance of a triple point in the size pressure plane. But the question is, if once L is fixed, this triple point appears in a pressure-density plane. And from Vega and Monson's article I do not see this interpretation clearly.
In the PRL from 2020, where in the SI the authors derive the law, they derive it from equation S3. But, in this equation, if q_i is considered constant, which is what happens in each of the thermodynamic systems studied, dq_i =0, and equation S3 becomes S1, which is the basis for justifying Gibbs' law in its classical formulation. I see a flaw in the reasoning here.
And reasoning by reductio ad absurdum, a sphere could be considered a particular case of an ellipsoid where the semi-axes are equal a=b=c= R. In the application of this generalised Gibbs law to the system of spheres+rods, Why not consider these two additional degrees of freedom for the spheres, and take into account as unfrozen only the length of the rod? Or consider as susceptible to change the dimensions perpendicular to the length of the rod?
That is, I think there is some arbitrariness in the range of variation of the interaction parameter M, but if it were to be considered in all its possibilities, itwould actually indicate that the Gibbs phase rule is almost without content, and indeed it does not apply limits for the number of phases at coexistence.
I stress that I do not want my arguments to be understood as reasons to reject the article. But I understand that the result the authors intend to use is sufficiently novel for the counterarguments I present here to be discussed.

4.- Some minor comments:
- In figure 1 is not indicated what are the size of the particles of phases diagramas in panels (a) an (b). I think this information is needed

- Panel (a) of Figure 1 shows the type of phase behavior for 2R_s_/D_r up to 3.5, but Figure 2 shows the phase diagrams for 2R_s_/D_r =12.5. It is not clear why Figure 1 shows only a narrow range, but subsequently the region explored in detail is outside this range.
And for my it is not clear if the information from fig 1 and table 1 is consistent with the shown in fig2. From Fig. 1 and Table 1 it would appear that the cases in Fig2 are in region I, II or III, but the triple regions reported in Fig2 do not appear to be the same as those indicated in Table 1. Maybe this can be clarified.

- Figure S1 (in the supplementary information) does not show the case with q=1. I believe this is the most studied case in the literature, and it may be interesting to see the predictions of the theory for this particular case.

- In my opinion the number of citations in the article is excessive, especially when most of them are introduced in the first paragraphs to discuss the state of the art. Perhaps the authors should try to reduce it, making a selection of the most relevant ones.

Reviewer: 2

Comments to the Author

This is a very valuable paper about the possible phase diagrams of mixtures of hard bodies, where the particles can be spherical, rod-like and plate-like. They have combined several theories to get the global phase diagrams. The reference one-component system is treated as accurately as possible, while the second component is taken into account with the help of Widom insertion method. They have tested the general phase rule on rod-sphere and rod-plate mixtures and showed that even 5 and 6 phases can be in phase coexistences. The have managed to explain the very complex phase behavior of rod-plate mixture of clay particles, too. I missed the mixture of plates and spheres in this paper, which would be a good playground with several experimental studies. However, I accept that plate-sphere mixture is worthy of another publication. The paper is very well-written and interesting. The results are scientifically meaningful for wide audience of colloidal science, physical chemistry and physics. I have only minor suggestions.

1) I think that the free energy of one component system is not complete, because the ideal free energy term should be phi ln(phi)-phi. Therefore I would check the equation after "The free energy of a system containing only component 1 contains both ideal and non-ideal contributions:"

2) The aspect ratio is usually defined as L/D instead of D/L in most of the earlier papers. It is widely accepted that L/D>1 for prolate and L/D<1 for oblate shapes. I would use L/D in this work for the aspect ratio, too. In the paper both definitions are used (see the figures and the text).

3) I do not understand why R_s/D_r is considered to be the size ratio on page 8. Why is it not D_s/D_r, which is actually the diameter ratio? I would introduce D_s instead of using 2*R_s.

4) I would explain the Gibbs phase rule of athermal systems, because the thermal one is written down in text books. I would say that instead of "2" there is "1" in the equation.

5) I would not say "isostructural" isotropic fluid-fluid phase transition, because the isotropic fluid cannot have different structures. In the case of solid, the isostructural solid-solid transition has meaning, because the solid can have different structures.

6) I like the conclusion very much, but I miss the possible ways to get over the challenging issues. I would mention that the combination of the cell-theory and the scaled particle theory can be a possible way to get better phase diagrams where both components treated as inhomogeneous fluids. In this path there are some attempts in the literature, which are not done for binary mixtures. See the papers of M. P. Taylor and H. Löwen.

Author's Response to Peer Review Comments:

Response to reviewers of: “Multi-Phase Coexistence in Binary Hard Colloidal Mixtures: Predictions from a Simple Algebraic Theory”.

Below are the comments from the reviewers and point by point responses from the authors (‘RA’). The response to the comments is shown in blue and additions/modifications made in the manuscript are shown in green. The line numbers indicated correspond to the file “Revised manuscript (highlighted).pdf” where we also highlighted any modifications and additions made to our submitted manuscript in blue and red. The reference numbers refer to the reference numbers in the revised manuscript without highlights “Revised manuscript.pdf”.

Reviewer: 1

Recommendation: This paper may be publishable, but major revision is needed; I would like to be invited to review any future revision.

Comments:

The authors present in this work a proposal to calculate the free energy in multicomponent fluids. This theory incorporates the possibility that one of the components (and only one) is arranged in structured phases, with positional and/or positional order, while the other component behaves as a fluid. The authors use this theory to predict the phase diagram in binary mixtures of rod-shaped (spherocylinder) and spherical particles and disk-shaped (cylinder) and spherical particles. Both mixtures are studied over a wide range of parameters. The obtained phase diagrams show regions with coexistence of various phase numbers. These coexistences are rationalized by using the so-called generalized phase Gibbs rule. This is a serious, deep and interesting work. It is well written, and the results are clearly presented. I believe that the theoretical approach can be very interesting for the study of a large number of systems. The results will undoubtedly be very interesting for a better understanding of the mixing of colloidal systems. However, I have serious doubts about the discussion of the results. I think this discussion needs further reflection, and probably some extra work. My comments are as follows:

RA: We thank the reviewer for the positive remarks on our manuscript and the useful comments and suggestions.

1: There is one aspect in the wording of the article that leads to confusion. In the introductory part of the article it is stated that " However, these studies do not include the formation of positionally ordered phases of both components simultaneously. In this study, we allow both components to adopt orientational and positional ordered states and we showcase a wide range of multi-phase coexistences that can be reconfigured by tuning the principal size ratios of the mixture." But further on, below equation 1 it is clarified that "It is important to note that the Widom expression66 is used for the chemical potential of component 2, which means that component 2 is assumed to behave as a fluid phase with no orientational or translational order. This means that, using this approach, it is not possible to account for binary crystals or biaxial phases where both components are ordered."
I understand this to mean that it is not possible to calculate the free energy of phases in which both components are ordered. This is a limitation of the theory, which does not introduce the possibility of highly ordered phases that may be the most stable at high packing. This limitation makes the theory not valid in all cases, even though it maintains an important range of applicability. But this sentence is contradictory to the previous sentence, which gives the theory a range of validity that the authors themselves admit it does not have. In fact, theories such as those disqualified by the authors in the previous paragraph are also capable of predicting situations where one of the components is ordered, and the other is a fluid. This aspect should be clarified by the authors, clarifying the range of applicability of their theory by indicating for which type of phases the theory can be applied. Here it is also important for the authors to point out the advantages of their theory over older theories. I think

the authors need to spend some time justifying why their proposal is something more than "just another theory" (if it is).

RA: We agree that the statement in our manuscript, as mentioned by the reviewer, is misleading. It is indeed not possible to calculate the free energy of a binary colloidal mixture in which both components are ordered simultaneously within the same phase state with our presented theory. The advantage of the theory with respect to previous theories is that we include phase states of colloidal mixtures where component 1 is positionally ordered and component 2 is disordered but also phase states where component 2 is positionally ordered and component 1 is disordered. Previous works focuses mostly on either one of these situations which limits the concentration and size range that can be considered for one of the two components and does not allow for coexistences between the two types of phases explained in the previous section. This is not the case for our work which makes it possible to provide an overview of possible phase coexistences for a wide range of size ratios and concentrations. Another advantage is the ease of use and the general applicability of the method which is highly valuable for experimentalists that want to determine phase diagrams for their parameters of interest.

About the limitation that we cannot incorporate a binary phase where both components 1 and 2 are ordered, we agree with the statement of the reviewer: “This is a limitation of the theory, which does not introduce the possibility of highly ordered phases that may be the most stable at high packing. This limitation makes the theory not valid in all cases, even though it maintains an important range of applicability.” At high packing fractions, such phases might indeed be the most stable. However, it is important to point out that our phase diagrams consistently feature strong demixing and fractionation effects. This means that phases coexisting at any of the multi-phase equilibria are all strongly enriched in one component while the other species is only present at relatively small volume fractions. Under such conditions, orientational or positional order of the minor component will have only a minor influence on the free energy of the binary phase. We further wish to reiterate that our purpose is to present a simple and algebraic theory that enables "a quick scan" of the phase diagram for a wide range of size and shape parameters without the need of tedious numerical computations. A more rigorous theoretical platform for dealing with strongly inhomogenous fluid mixtures would be classical density functional theory (DFT) [see e.g. Mederos et al. for a discussion on hard-particle mixtures]. Even though DFT would, at least in principle, enable both components to be fully ordered both orientationally and positionally, such theories often suffer from significant approximations such as frozen-in orientational order (leading to a poor representation of the orientational entropy) while the numerical burden associated with resolving the minmization conditions can be a strong impediment to a wide-range exploration of the phase diagrams that we have sought to pursue here.

To clarify the misleading statement mentioned by the reviewer and to emphasize the advantage, novelty and limitations of our presented theory, the text in lines 56–59 was changed to:
However, these studies only include positionally ordered phases of one component. This imposes a limitation on the range of applicability with regards to size ratios and concentrations and does not allow computation of coexistence between a phase where one component exhibits positional order and a phase where the other component exhibits positional order. In this study, we overcome this limitation and include phases where either one of the components is allowed to adopt orientational and positional ordered states and we showcase a wide range of possible multi-phase coexistences that can be reconfigured by tuning the principal size ratios of the mixture.

With the general theoretical method outlined in this Letter, it is not possible to account for phase states where both components are ordered simultaneously. It is important to point out that our phase diagrams consistently feature strong demixing and fractionation effects and the phase states coexisting at any of the multi-phase equilibria are all strongly enriched in one component while the other species is only present at relatively small volume fractions. Under such conditions, orientational or positional order of the minor component will have only a minor influence on the free energy of any

phase state of the binary mixture. In dense mixtures with different types of anisotropic particles, there are likely conditions under which both components remain well-mixed and generate structures in which both components are ordered. Theoretically, these cases could be addressed using density functional theory (see Mederos et al.[47] for a discussion on dense multi-component hard-particle fluids), although treating both components as freely-rotating objects poses serious technical difficulties.[48,49] For future work it will be of interest to compare the relative stability of phase states in which both components exhibit order with respect to the (multi-phase) demixing regions discussed here.

2: One of the main limitations of the article is that the authors present little evidence of the accuracy of their theory. A qualitative comparison is made with the experimental phase diagram published by Van de Kooij and Lekkerkerkerker. As the authors point out, these experimental data are obtained from a system with a certain level of polydispersity, which makes a direct comparison doubtful. In contrast, no comparison with simulation results is made or discussed. It is true that it can be difficult to find simulation results with complete phase diagrams as calculated by the authors. But there are published in the literature phase diagrams with some of the transitions indicated, especially at low packing. Moreover, from the theory it is possible to obtain, in addition to the phase diagram, the equation of state in each of the phases found. The information on these equations of state for the systems studied in many of the phases is available in the literature, and if necessary can be calculated with not very complicate Monte Carlo simulations. It would be interesting for the authors to compare their theoretical predictions with these EOS obtained by simulation, and also to indicate whether this comparison between simulation and theory is better than in the case of other theories. I think it is something accessible, and it would give information on the range of validity of the proposed theory, and the improvement over previous works.

RA: We understand the reviewer's opinion that the lack of comparison with simulations and experiments is a limitation of our manuscript. However, it should be clarified that the main selling point of our presented theory is not necessarily an improvement over previous theories mentioned in our introduction but that it has a wider range of applicability which allows to determine the overview of all possible coexistences between the phases considered as shown in the manuscript (Fig. 2a). As mentioned in line 119 of our revised manuscript, the free energy expression used for the binary mixture, Eq. 1, can be seen as a canonical equivalent of the recently improved free volume theory (FVT) method for colloidal mixtures which only incorporates ordered phases for one of the components. In references 14, 46 and 56, the validity of this FVT method has been confirmed with experiments and simulations for various colloidal mixtures. In this manuscript we chose to highlight the novelty of the newly proposed method as a letter by showing the possibility to take into account positionally ordered phases for both components allowing to predict an overview for the phase behaviour of colloidal mixtures for a wide range of parameters, which has received little attention, instead of making a detailed comparison between the results and simulation/experimental work.

Furthermore, most simulation (and experimental) work that we were able to find for rod/sphere mixtures focuses only on one of the regions indicated in Fig. 1a and only on the low-density region. Although these simulation results are generally in line with the theoretical predictions of Fig. 1a, the novelty of our results does not lie in this range of parameters. With our work we highlight the wide range of possible coexistences and in which parameter space they can be expected. It would therefore be more interesting to see if the predicted changes in phase diagram morphology as a function of size ratio matches with simulations. However, we did not manage to find such simulation results. Furthermore, we also believe there is a lack of EOS for binary colloidal mixtures obtained with simulations. For future work it is of interest to provide a more complete overview of theoretical, simulation and experimental results on colloidal mixtures. However, we believe this is not appropriate for the letter format of the current journal and we plan to write an additional manuscript focusing on this topic in the near future.

We added the following text to the manuscript (lines 126–131):
"The FVT predictions were shown to be in qualitative and reasonable quantitative agreement with computer simulation and experimental results which is an indication of the validity of Eq. 1 in the parameter range of these studies. In the future, a detailed comparison between the predicted variety of possible phase coexistences presented in this Letter and an overview of computer simulation results and experimental observations on various colloidal mixtures is desirable."

3: The authors propose a modification to such a classical result as the Gibbs phase law. The truth is that I am not entirely comfortable with this proposal, with the so-called generalised Gibbs phase law. This modification to the phase law was originally proposed by Vega and Monson, and then used (and analysed) by the authors in earlier publications. With these bibliographical precedents, and without having solid arguments to prove its invalidity, I understand that it is not my role as a reviewer to point out my doubts as a reason for not publishing the article. On the contrary, although I may find it shocking, since there are simulation results that seem to support the authors' interpretation,I believe that the possible controversy is a reason for publication, so that the community can discuss and pronounce itself on the subject. But I do not want to fail to point out here my doubts. The main one is that I understand that the extension of Gibbs' law proposed by the authors would only make sense if the degrees of freedom associated with the dimensions of the particles were not frozen, if they could change. And this is not the case, since every system under study has fixed particle dimensions. I mean, although the study is extended for different sizes of spheres, rods and disks, each phase diagram is calculated for fixed values of these dimensions. In Vega and Moson's article, this rule is used to explain the appearance of a triple point in the size pressure plane. But the question is, if once L is fixed, this triple point appears in a pressure-density plane. And from Vega and Monson's article I do not see this interpretation clearly.

RA: The application of the Gibbs phase law to multi-phase coexistences in colloidal systems is indeed a matter of debate. The discussion on using the generalized Gibbs phase rule is however not the main topic of this manuscript. We do believe our manuscript provides further support for the use of the generalized Gibbs phase rule to predict the maximum number of coexisting phases in a colloidal mixture. It is indeed counter-intuitive to look at the shape of a colloidal particle as a parameter that influences its interaction since the shape of the particles is "frozen". However, the shape of the particle does affect the relative stability of the considered phases and therefore, although it is not a variable that can change in conventional experiments, it can be expected that there are specific values for the shape parameter of a particle where multiple phase coexistence regions meet, and a multi-phase coexistence region is found. Therefore, multi-phase coexistence regions with more than 3 phases only occur for very specific shape parameters and finding those exact parameters is unlikely with simulations or experiments because indeed it is challenging to systematically vary the shape of a particle. But theoretically these multi-phase coexistence regions do exist and they are also found in experiments as highlighted by the results of van der Kooij and Lekkerkerker (Fig. 3b). The reason why they are found in experiments, even though the size and shape of the particles does not exactly correspond to the theoretically predicted parameters, is because the particles have some polydispersity and therefore multi-phase coexistence regions can be expected in the regions in the parameter-space close to the exact theoretically predicted parameters. This is one of the reasons why predicting the parameters for such multi-phase coexistence regions is of importance and why we believe that the relevance of the generalized Gibbs phase rule applied to colloidal mixtures should be acknowledged, even though it still is a controversial topic.

In the PRL from 2020, where in the SI the authors derive the law, they derive it from equation S3. But, in this equation, if q_i is considered constant, which is what happens in each of the thermodynamic systems studied, dq_i =0, and equation S3 becomes S1, which is the basis for justifying Gibbs' law in its classical formulation. I see a flaw in the reasoning here.

And reasoning by reductio ad absurdum, a sphere could be considered a particular case of an ellipsoid where the semi-axes are equal a=b=c= R. In the application of this generalised Gibbs law to the system of spheres+rods, Why not consider these two additional degrees of freedom for the spheres, and take into account as unfrozen only the length of the rod? Or consider as susceptible to change the dimensions perpendicular to the length of the rod? That is, I think there is some arbitrariness in the range of variation of the interaction parameter $\mathcal{M}$, but if it were to be considered in all its possibilities, itwould actually indicate that the Gibbs phase rule is almost without content, and indeed it does not apply limits for the number of phases at coexistence. I stress that I do not want my arguments to be understood as reasons to reject the article. But I understand that the result the authors intend to use is sufficiently novel for the counterarguments I present here to be discussed.

RA: Spheres can indeed be viewed as ellipsoids with specific shape parameters. With our method it would be possible to take into account additional degrees of freedom for the spheres and consider them as ellipsoids with additional degrees of freedom and in that case coexistences between more than five phases, such as seen for plate/rod mixtures in Fig. 3c, should be expected for specific shape parameters. The overview presented in Fig. 1a is just one slice out of the full overview of possible phase coexistences between rods and ellipsoids.

In the manuscript we provide a brief "derivation" of the generalized Gibbs phase rule and show that it follows naturally when considering the number of parameters in the system and the number of equations that must be solved to find coexistence densities. The parameter $\mathcal{M}$, purely defined by the number of parameters that affect an EOS, and the generalized Gibbs phase rule do apply the limit to the maximum number of coexisting phases since the number of equations that have to be solved cannot be larger than the number of parameters in the equations.

In the manuscript, we added the following sentence (lines 194–198) for clarity:
"Therefore, even though the shapes and sizes of particles are usually not variable parameters in conventional colloidal systems, there are specific values for size ratios and shape parameters where additional phases meet the coexistence conditions and coexistences between more than three phases are theoretically possible even in athermal two-component systems."

4: Some minor comments:
- In figure 1 is not indicated what are the size of the particles of phases diagramas in panels (a) an (b). I think this information is needed

RA: Panels (b) and (c) of figure 1 are phase diagrams and they belong to the size and shape parameters indicated by the symbols (quintuple points) in panel (a) as mentioned in the caption. The exact values were indeed not given but we added them to the caption.

- Panel (a) of Figure 1 shows the type of phase behavior for 2R_s_/D_r up to 3.5, but Figure 2 shows the phase diagrams for 2R_s_/D_r =12.5. It is not clear why Figure 1 shows only a narrow range, but subsequently the region explored in detail is outside this range.
And for my it is not clear if the information from fig 1 and table 1 is consistent with the shown in fig2. From Fig. 1 and Table 1 it would appear that the cases in Fig2 are in region I, II or III, but the triple regions reported in Fig2 do not appear to be the same as those indicated in Table 1. Maybe this can be clarified.

RA: In figure 1 and table 2 we did not include multi-phase coexistence regions that contain two phases with the same structure such as the isotropic-isotropic coexistence discussed in Fig. 2. We clarified this in the caption and in the main text (lines 169–171):

"Coexistence regions that contain two phases with the same structure but different concentrations, such as N–I–I–FCC coexistence, are not shown in the overview of Fig. 1a and Table 2".

- Figure S1 (in the supplementary information) does not show the case with q=1. I believe this is the most studied case in the literature, and it may be interesting to see the predictions of the theory for this particular case.

RA: With Figure S1, we chose to show one representative phase diagram for each of the indicated regions in Fig. 1a (plus 1 extra for a large q to show the increase of the I-FCC region when the spheres become relatively large) and chose a size ratio of q=1.5 for regions IV and IX since it is more in the center of the region. We replaced these two phase diagrams with the ones for colloidal mixtures that have a size ratio of q=1. We also added a statement to the section "Supporting Information Available" (line 335) that the Mathematica notebooks used for the calculations in this Letter are available on request so that readers can easily calculate phase diagrams for other parameters if desired.

- In my opinion the number of citations in the article is excessive, especially when most of them are introduced in the first paragraphs to discuss the state of the art. Perhaps the authors should try to reduce it, making a selection of the most relevant ones.

RA: We agree with the reviewer that finding the most relevant citations is difficult with the large number of citations in the manuscript and we chose to remove a selection of citations (see the citations highlighted in red in the file "Revised manuscript (highlighted).pdf").

We also chose to remove some small sections of text to reduce the length of our manuscript to compensate for some of the additions made during the revision stage (lines 27, 45-49, 206-208, 265-267).

Additional Questions:
Urgency: Moderate
Significance: High
Novelty: High
Scholarly Presentation: High
Is the paper likely to interest a substantial number of physical chemists, not just specialists working in the authors' area of research?: Yes

Reviewer: 2

Recommendation: This paper is publishable subject to minor revisions noted. Further review is not needed.

Comments:

This is a very valuable paper about the possible phase diagrams of mixtures of hard bodies, where the particles can be spherical, rod-like and plate-like. They have combined several theories to get the global phase diagrams. The reference one-component system is treated as accurately as possible, while the second component is taken into account with the help of Widom insertion method. They have tested the general phase rule on rod-sphere and rod-plate mixtures and showed that even 5 and 6 phases can be in phase coexistences. The have managed to explain the very complex phase behavior of rod-plate mixture of clay particles, too. I missed the mixture of plates and spheres in this paper, which would be a good playground with several experimental studies. However, I accept that plate-sphere mixture is worthy of another publication. The paper is very well-written and interesting. The results

are scientifically meaningful for wide audience of colloidal science, physical chemistry and physics. I have only minor suggestions.

RA: We thank the reviewer for their careful assessment of our manuscript and the positive recommendation.

1: I think that the free energy of one component system is not complete, because the ideal free energy term should be phi ln(phi)-phi. Therefore I would check the equation after "The free energy of a system containing only component 1 contains both ideal and non-ideal contributions:"

RA: This was indeed incorrect and we corrected the equation in the manuscript (line 97). Many thanks to the reviewer for noting this.

2: The aspect ratio is usually defined as L/D instead of D/L in most of the earlier papers. It is widely accepted that L/D>1 for prolate and L/D<1 for oblate shapes. I would use L/D in this work for the aspect ratio, too. In the paper both definitions are used (see the figures and the text).

RA: The reason we chose D/L for Figure 1 is to use the same representation of the phase overview as used for rod/polymer mixtures in reference 74. For clarification we now mention inverse aspect ratio when we discuss figure 1.

3: I do not understand why R_s/D_r is considered to be the size ratio on page 8. Why is it not D_s/D_r, which is actually the diameter ratio? I would introduce D_s instead of using 2*R_s.

RA: We changed 2*R_s to D_s throughout the manuscript.

4: I would explain the Gibbs phase rule of athermal systems, because the thermal one is written down in text books. I would say that instead of "2" there is "1" in the equation.

RA: For clarity we added the following sentence (line 180):
The term +1 can be replaced with +2 to describe systems with variable temperature.

5: I would not say "isostructural" isotropic fluid-fluid phase transition, because the isotropic fluid cannot have different structures. In the case of solid, the isostructural solid-solid transition has meaning, because the solid can have different structures.

RA: We removed the term isostructural when discussing isotropic-isotropic coexistence because it does not add further information.

6: I like the conclusion very much, but I miss the possible ways to get over the challenging issues. I would mention that the combination of the cell-theory and the scaled particle theory can be a possible way to get better phase diagrams where both components treated as inhomogeneous fluids. In this path there are some attempts in the literature, which are not done for binary mixtures. See the papers of M. P. Taylor and H. Löwen.

We agree that a combination of cell theory and scaled particle theory might be a possible solution for part of the challenging issues discussed in the manuscript. One of these issues is that the ordering of the main component is not accounted for in the free volume description for the minor component. This could be improved by determining the free volume available for the minor component by a combination of two separate contributions: One based on the effective free volume available in the crystal of the pure main component based on a cell theory approach and one that accounts for the interactions between the particles of the minor components within this effective free volume based

on a scaled particle theory approach. This method has for example been used in references 56 and 70 for phases where spheres are the minor component. To apply this method to phases where the minor component is anisotropic would require to take into account the reduced orientational freedom in a way similar to the methods used by Taylor and Löwen. For specific phases, other approximations have also resulted in reasonable improvements over the pure scaled particle theory approach for the minor component. Although these methods can result in improvements for specific phases, it is not feasible to apply in a broadly applicable theoretical method as discussed in our manuscript. Furthermore, incorporating phases in which both components are positionally ordered simultaneously will require methods beyond scaled particle theory.

We made a brief statement in our manuscript in lines 148—154 about this issue and now also added a sentence to the conclusions to highlight this possible method to improve approximations made in our general theory (lines 312–314):
"For ordered phases of specific colloidal mixtures, a combination of cell theory and scaled particle theory[56,70] can potentially be applied to improve the predictions for the work of insertion for the minor component."

Additional Questions:
Urgency: High
Significance: High
Novelty: High
Scholarly Presentation: High
Is the paper likely to interest a substantial number of physical chemists, not just specialists working in the authors' area of research?: Yes

jz-2022-03138x.R2

Name: Peer Review Information for "Multi-Phase Coexistence in Binary Hard Colloidal Mixtures: Predictions From a Simple Algebraic Theory"

Second Round of Reviewer Comments

Reviewer: 1

Comments to the Author
The answers provided by the authors, and their discussion of my doubts and comments are correct, and very valuable. I understand their arguments and nuances introduced due to my first two comments.

I am still unclear about the answer to my doubts about the validity and applications of an alleged generalized Gibbs rule. As I said in my previous report, I understand that, given the experimental evidence and previous publications, this is not a reason to reject the article. Moreover, the arguments provided, and the modifications added, are valuable. I understand that, despite my scepticism, this is an aspect that deserves to be submitted to the evaluation of the whole community.

One minor issue. I detect what I think is a typo, the dimensions for the particles introduced in the caption of figure 1b and 1c I think are incorrect, that the ratio of diameters is exchanged with Dr/Lr, or something like that. I suggest the authors to check it.

Author's Response to Peer Review Comments:

Response to reviewer of: “Multi-Phase Coexistence in Binary Hard Colloidal Mixtures: Predictions from a Simple Algebraic Theory”. (Second round of review.)

Below are the comments from the reviewer and responses from the authors (‘RA’).

Reviewer: 1

Recommendation: This paper is publishable subject to minor revisions noted. Further review is not needed.

Comments:

The answers provided by the authors, and their discussion of my doubts and comments are correct, and very valuable. I understand their arguments and nuances introduced due to my first two comments.

I am still unclear about the answer to my doubts about the validity and applications of an alleged generalized Gibbs rule. As I said in my previous report, I understand that, given the experimental evidence and previous publications, this is not a reason to reject the article. Moreover, the arguments provided, and the modifications added, are valuable. I understand that, despite my scepticism, this is an aspect that deserves to be submitted to the evaluation of the whole community.

RA: We thank the reviewer for the positive remarks on our provided answers and revisions made in the first round of reviews.

One minor issue. I detect what I think is a typo, the dimensions for the particles introduced in the caption of figure 1b and 1c I think are incorrect, that the ratio of diameters is exchanged with Dr/Lr, or something like that. I suggest the authors to check it.

RA: This is indeed a typo and the ratio Dr/Lr should be Ds/Dr and vice versa, as mentioned by the reviewer. We corrected this error in the revised manuscript (page 9 in “Manuscript 2nd revision (highlighted).pdf”, caption of Figure 1). We are thankful of the reviewer for spotting this error.

Additional Questions:
Urgency: Moderate
Significance: High
Novelty: High
Scholarly Presentation: High
Is the paper likely to interest a substantial number of physical chemists, not just specialists working in the authors' area of research?: Yes
